# Investigation into the underlying regulatory mechanisms shaping inflorescence architecture in *Chenopodium quinoa*

**DOI:** 10.1186/s12864-019-6027-0

**Published:** 2019-08-17

**Authors:** Qi Wu, Xue Bai, Wei Zhao, Xiaodong Shi, Dabing Xiang, Yan Wan, Xiaoyong Wu, Yanxia Sun, Jianglin Zhao, Lianxin Peng, Gang Zhao

**Affiliations:** 1 Key Laboratory of Coarse Cereal Processing Ministry of Agriculture and Rural Affairs, College of Pharmacy and Biological Engineering, Chengdu University, Chengluo road 2025, Shiling town, Longquanyi District, Chengdu, 610106 Sichuan province People’s Republic of China; 2 National Research and Development Center for Coarse Cereal Processing, College of Pharmacy and Biological Engineering, Chengdu University, Chengdu, 610106 People’s Republic of China

**Keywords:** *Chenopodium quinoa*, Inflorescence architecture, RNA-seq, Transcriptome analysis, Weighted gene co-expression network analysis

## Abstract

**Background:**

Inflorescence architecture is denoted by the spatial arrangement of various lateral branches and florets formed on them, which is shaped by a complex of regulators. Unveiling of the regulatory mechanisms underlying inflorescence architecture is pivotal for improving crop yield potential. Quinoa (*Chenopodium quinoa* Willd), a pseudo cereal originated from Andean region of South America, has been widely recognized as a functional super food due to its excellent nutritional elements. Increasing worldwide consumption of this crop urgently calls for its yield improvement. However, dissection of the regulatory networks underlying quinoa inflorescence patterning is lacking.

**Results:**

In this study, we performed RNA-seq analysis on quinoa inflorescence samples collected from six developmental stages, yielding a total of 138.8 GB data. We screened 21,610 differentially expressed genes (DEGs) among all the stages through comparative analysis. Weighted Gene Co-Expression Network Analysis (WGCNA) was performed to categorize the DEGs into ten different modules. Subsequently, we placed emphasis on investigating the modules associated with none branched and branched inflorescence samples. We manually refined the coexpression networks with stringent edge weight cutoffs, and generated core networks using transcription factors and key inflorescence architecture related genes as seed nodes. The core networks were visualized and analyzed by Cytoscape to obtain hub genes in each network. Our finding indicates that the specific occurrence of B3, TALE, WOX, LSH, LFY, GRAS, bHLH, EIL, DOF, G2-like and YABBY family members in early reproductive stage modules, and of TFL, ERF, bZIP, HD-ZIP, C2H2, LBD, NAC, C3H, Nin-like and FAR1 family members in late reproductive stage modules, as well as the several different MADS subfamily members identified in both stages may account for shaping quinoa inflorescence architecture.

**Conclusion:**

In this study we carried out comparative transcriptome analysis of six different stages quinoa inflorescences, and using WGCNA we obtained the most highly potential central hubs for shaping inflorescence. The data obtained from this study will enhance our understanding of the gene network regulating quinoa inflorescence architecture, as well will supply with valuable genetic resources for high-yield elite breeding in the future.

**Electronic supplementary material:**

The online version of this article (10.1186/s12864-019-6027-0) contains supplementary material, which is available to authorized users.

## Background

Crop yield is mainly determined by three major factors: grain number per panicle, effective panicle number, and seed setting rate [1]. Under limited growth area increasing the grain number per panicle or individual plant is a fundamental and proficient way to improve the yield. This goal could be achieved via improving the inflorescence architecture. Inflorescence architecture refers to the space arrangement of florets and branches that ultimately form the grains. It is shaped by a complex of post-embryogenesis developmental processes [2–5]. Variation of inflorescence architecture occurs among different species [4]. In *Arabidopsis thaliana* after transition from vegetative phase to reproductive phase, a shoot apical meristem (SAM) converts into a main inflorescence meristem (IM). Subsequently, the IMs bear axillary meristems (AMs) that sequentially form floral meristems (FMs) or indeterminate branch meristems (BMs) [4, 6, 7]. The growth of an IM will stay indefinite before senescence. By contrast, in grass species such as rice (*Oryza sativa*), maize (*Zea mays*) and oat (*Avena sativa*), the AMs on the main IM produce primary (pBMs), secondary (sBMs) or high-order branch meristems on which spikelet meristems (SMs) and FMs are ultimately formed [6, 7]. The growth of grass IM is definite and terminated by flowers. Hence, where and when the BMs and FMs/SMs are formed along an IM, in other word, the identities and activities of various meristems can directly influence the inflorescence morphology [4, 5]. Over past few decades, combination of plant morphology, genetics and molecular biology approaches has unveiled many genes involved in those events, which significantly contributes to our understanding of the inflorescence architecture regulatory networks.

The classical *CLAVATA* (*CLV*)-*WUSCHEL* (*WUS*) feed-back regulatory loop is indispensable for maintaining the activity of pluripotent stem cells in the central region of SAM, which guarantees the potential to continuously generate leaf primordia or reproductive organs [4, 6, 7]. *clv1*, *clv2* and *clv3* mutants all display enlarged inflorescence size and increased floral organs due to overproliferation of stem cells [8–10]. Similarly, many evidences showed that mutations in rice *CLV1* homolog *FLORAL ORGAN NUMBER 1* (*FON1*) [11], *CLV3* homolog *FON4/2* [12], and in maize *CLV1* homolog *THICK TASSEL DWARF 1* (*TD1*) [13], *CLV2* homolog *FASCIATED EAR 2* (*FEA2*) [14] led to increased floral organ number and inflorescence size. The homeodomain-containing transcription factor (TF) WUS promotes the expression of *CLV3* which encodes a small CLV3/ESR-related (CLE) peptide [15, 16]. In turn *CLV3* represses the expression *WUS*. Another homeodomain TF-Class 1 KNOTTED 1-like homeobox (KNOX), which sophisticatedly controls the homeostasis between gibberellin and cytokinin, is also essential for establishment and maintainence of SAM [17]. The corresponding *KNOX* mutants in rice (*Oryza sativa HOMEOBOX* 1, *OSH1*) [18], maize (*KNOTTED 1*, *KN*1) [19], and *Arabidopsis* (*SHOOT MERISTEMLESS*, *STM*) [20] are defective in inflorescence morphology.

At optimal time the florigen proteins move from leaves to SAM to trigger downstream flowering programs, thus acquiring IM identity [3]. This process is very important for panicle structure and the ultimate grain yield in grass crops. Several rice MADS-box genes including *OsMADS14*, *OsMADS15*, *OsMADS18*, and *OsMADS34* (also called *PANICLE PHYTOMER 2*, *PAP2*) are demonstrated to play crucial roles in IM specification [21, 22]. Interestingly, their *Arabidopsis* counterparts such as *APETALA 1* (*AP1*) and *SEPALLATA* (*SEP*)-like MADS-box genes are responsible for floral organ identity determination [23–25], indicating divergent roles of those homologs between different species. The number of BMs produced on the IMs determines the final inflorescence branches [5]. The phosphatidylethanolamine-binding protein (PEBP) encoding gene *TERMINAL FLOWER 1* (*TFL1*) antagonizes with *AP1* and *LEAFY* (*LFY*) to coordinately modify inflorescence branching [23]. Overexpression of the rice *TFL1* homologs *REDUCED CULM NUMBER 1* (*RCN1*) and *RCN2* [26], or *Zea mays* homologs *CENTRORADIALIS 1* (*ZCN1*) to *ZCN6* [27] delays the conversion from IMs or BMs to FMs, thereby generating highly branched inflorescences. Consistently, the *Arabidopsis tfl1* mutant generates a determinate inflorescence [28]. Conversely, LFY, a plant-specific TF, acts upstream of and promotes the expression of *AP1* to specify FMs determination via suppression of *TFL1* [23]. Loss of function of *LFY* homologs results in hampered floral identify acquisition in *Arabidopsis* and *Petunia* [29]. Besides, some other MADS-box genes, including *SHORT VEGETATIVE PHASE* (*SVP*), *SUPPRESSOR OF OVEREXPRESSION OF CONSTANS 1* (*SOC1*), *AGAMOUS-LIKE 24* (*AGL24*) and *SEP4*, interact with *AP1* and function redundantly to repress the expression of *TFL1* [30]. Knockdown of rice *SVP*/*AGL24* homologs (*OsMADS22*, *OsMADS47*, and *OsMADS55*) and *SOC1* homologs (*OsMADS50*, *OsMADS56*) in *OsMADS34* (homolog of *SEP4*) mutant background leads to significantly enhanced branching panicle which mimics the phenotypes of *RCN* overexpressors [30]. OsSPL14, a SQUAMOSA promoter binding protein-like (SBP) box TF responsible for a rice yield quantitative trait loci (QTL) *Ideal Plant Architecture 1* (*IPA1*)/*Wealthy Farmer’s Panicle* (*WFP*), positively regulates the primary branch number and yield [31]. The maize SBP TF-TASSELSHEATH4 (TSH4) acts downstream of meristem identity genes including *RAMOSA1* (*RA1*), *RA2*, *RA3* and *BRANCHED SILKLESS*1 (*BD1*) to modify the growth of lateral branches [32].

The number of SMs/FMs formed on IMs largely influences the final grain yield [5], and this developmental process is regulated by a series of key genes. The rice *UNUSUAL FLORAL ORGAN* (*UFO*) homolog *ABERRANT PANICLE ORGANIZATION 1* (*APO1*) depresses transition from IMs to SMs [33]. Similarly, the interacting protein of APO1, APO2/RICE FLORICAULA (RFL) /OsLFY, acts as a suppressor for the transition from IMs to FMs in rice, and the corresponding mutants harbor decreased branched panicle [34]. Remarkably, the functions of rice *APO1* and *APO2/RFL* are distinguished from their *Arabidopsis* counterparts which are positive determinants for floral organ specification. Further investigation indicates the divergent roles of *APO1* and *APO2/RFL* homologs between different species may arise from the distinct expression patterns and functional modes of *LFY* genes. Recently, accumulating studies indicate that the Arabidopsis LSH1 and Oryza G1 (ALOG) family proteins are vital modulators of inflorescence structure. TAWAWA1 (TWA1) is a rice nuclear-localized ALOG protein [35]. It prolongs the indeterminate status of IMs to limit the formation of SMs by inducing the expression of *SVP*-*like* genes (such as *OsMADS22*, *OsMADS47*, and *OsMADS55*) while depressing the floral organ formation related genes (such as *OsMADS7*, *OsMADS3*, and *OsMADS58*) [35]. Up-regulation of *TWA1* gives rise to highly-branched panicle whereas down-regulation of *TWA1* leads to an opposite phenotype [35]. The tomato (*Solanum lycopersicum*) *TWA1* homolog *TERMINATING FLOWER* (*TMF*) functions in a similar manner as does in rice, and *tmf* mutants produce single flower-terminated primary branches rather than sympodial inflorescences [36]. Furthermore, Transition from SMs to FMs is also closely associated with the final panicle structure [5]. Evidences obtained from multiple grass species indicate that ethylene-response factor (ERF)-class AP2 TFs act as pivotal mediators for this process. For instance, rice *FRIZZY PANICLE* (*FZP*)/*BRANCHED FLORETLESS 1* (*BFL1*) [37], *SUPERNUMERARY BRACT* (*SNB*) [38] and *MULTI-FLORET SPIKELET 1* (*MFS1*) [39], maize *BD1* and *INDETERMINATE SPIKELET 1* (*IDS1*) [40], and *Brachypodium distachyon MORE SPIKELETS 1* (*MOS1*, [41]) are all essential for triggering FMs specification.

The knowledge acquired from model plants provides significant insights into the inflorescence formation and could be appropriately adopted for investigating and improving the crops yield. Quinoa (*Chenopodium quinoa*) is an ancient pseudocereal of *Amaranthaceae* family originating from Andean region in South America [42]. It has been widely recognized as an extraordinary “superfood” because of its abundant and excellent-balanced nutritional components [43], which is especially highlighted by declaration of 2013 as the International Year of Quinoa by United Nations [44]. From the beginning of domestication and cultivation of quinoa in about 5000~7000 years ago, to date growing of quinoa has expanded to more than 100 countries [45]. However, the worldwide rapid increasing consumption of quinoa urgently demands more breeding efforts to improve the yield-related agronomic traits [44, 45]. Deciphering and understanding of the molecular mechanisms shaping quinoa inflorescence architecture is necessary and helpful to fulfill this purpose. Previously, a few studies have well detailed the sequential changes in quinoa inflorescence morphology [46]. There are two major types of inflorescence architecture in quinoa: Type I: Glomerulate inflorescence; Type II: amaranthiform inflorescence. After floral transition, the inflorescence arises from the apical of the plant or from leaf axils along the stem. As the inflorescence elongates, the secondary branches are formed along with the main axis. As growth goes on, a group of florets may form directly on the secondary branches (Type II), or form on the tertiary branches which are born on the secondary branches (Type I). Thus, Type II inflorescence lacks tertiary branches compared with Type I. The timing of branching (secondary and tertiary) and formation of florets determine the ultimate inflorescence structure. The preliminary studies have enhanced our understanding of the quinoa inflorescence morphogenesis, however, investigation regarding to the in-depth regulatory networks is lacking. Due to the unavailability of quinoa genome sequences before 2017, dissecting the genetic basis by quantitative trait locus (QTL) mapping is limited. Compared with the many evidences related to inflorescence patterning found in model plants such as *Arabidopsis* and rice, to our knowledge, up to date no genes have been discovered to participate in quinoa inflorescence patterning.

The advances of high-throughput next generation sequencing (NGS) technology in analyzing global transcription regulation combined with the recently released high-quality quinoa reference genome provides us a good opportunity to explore the complexity of transcription regulation shaping inflorescence. In the present study, we performed the first attempt to decipher the molecular basis of quinoa inflorescence regulation. We carried out comparative transcriptome analysis of quinoa inflorescence samples collected from six different developmental stages, and then we generated core transcriptional regulatory networks using Weighted Gene Co-Expression Network Analysis (WGCNA) method and identified several sets of potential candidates regulating quinoa inflorescence structure. The data obtained from this study is very helpful to enhance our understanding of the gene network regulating quinoa inflorescence architecture, and also it will provide highly potential candidates which could be manipulated for elite cultivars breeding in the future.

## Results

### RNA sequencing of different-stage quinoa inflorescences tissues

**Fig. 1:**
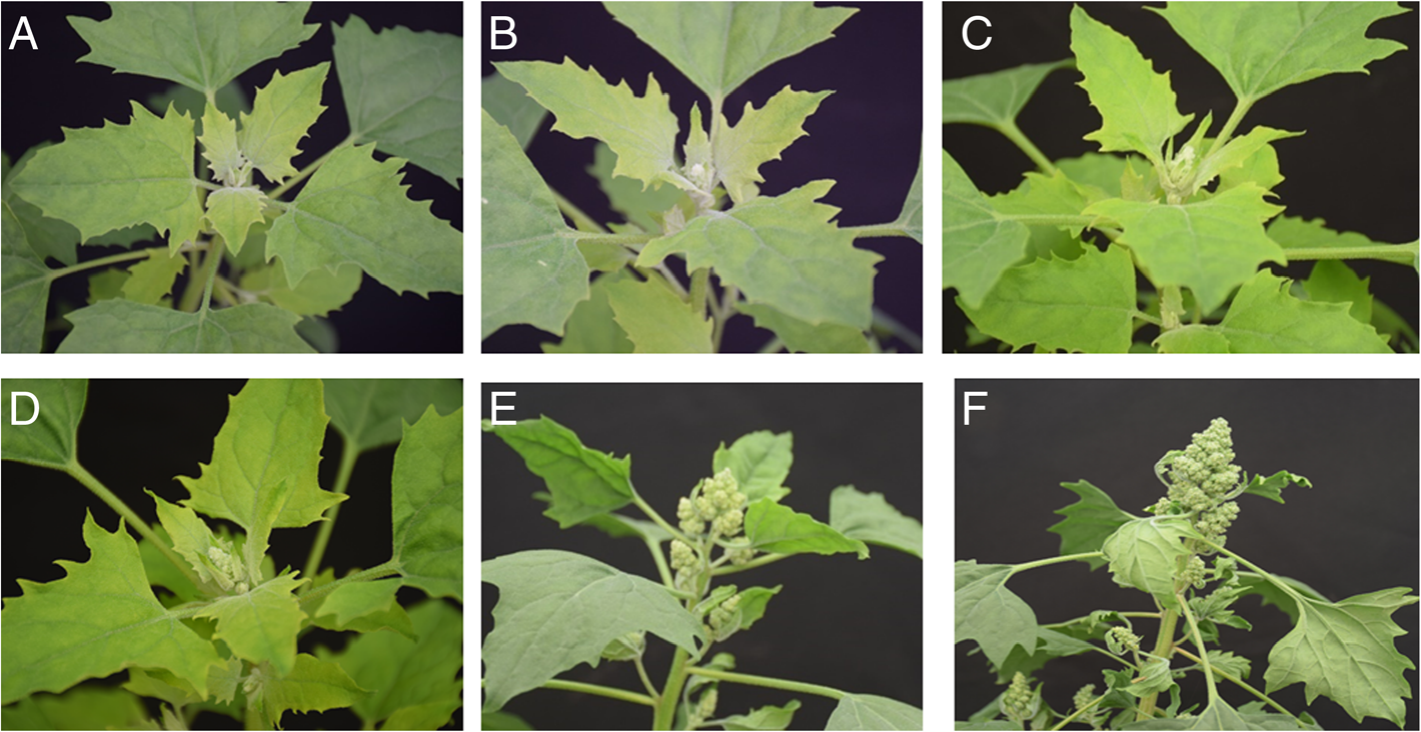
The morphologies of quinoa inflorescence at different developmental stages. The quinoa seeds were sown on September 16th (**a**), September 9th (**b**), September 2nd (**c**), August 24th (**d**), July 29th (**e**), and July 19th (**f**), 2017. The quinoa plants were grown under nature photoperiod with regular management in a greenhouse at Chengdu University. The inflorescence samples were collected on December 15th, 2017 and were denoted as YP1 (**a**), YP2 (**b**), YP3 (**c**), YP4 (**d**), P1 (**e**) and P2 (**f**) for RNA sequencing

The quinoa variety “LL-1” plants used in this study were grown in a greenhouse in Chengdu University. “LL-1” is a Type I inflorescence quinoa. The inflorescence samples were collected on December 15th, 2017 from three to five quinoa plants which were sown on July 19th, July 29th, August 24th, September 2nd, September 9th and September 16th, 2017, respectively referring to as 149 days after sowing (DAS), 139 DAS, 113 DAS, 104 DAS, 97 DAS and 90 DAS (Fig. 1). Sampling was performed with three biological replicates for each time point. YP1 (young panicle), YP2, YP3, YP4, P1 (elder panicle) and P2 (Fig. 1, a-f) were used to denote for the materials ranging from early reproductive to late reproductive stages. At phase YP1 (Fig. 1, a), the floral bud did not penetrate through the bracts, but actually the small floral bud was visible when manually removing the surrounding bracts. At YP1 the plants had just finished floral transition, apical meristem was changed to young inflorescence, the branches have not been formed (Fig. 1, a). At YP2, the main inflorescence began to elongate, and the secondary branches began to form (Fig. 1, b). At YP3, the lateral secondary branches were shortly elongated (Fig. 1, c). At YP4, the lateral secondary branches were more elongated and the tertiary branches began to form (Fig. 1, d). At P1, the secondary and tertiary branches were largely elongated and the florets on the lateral branches were formed (Fig. 1, e). At P2, the branches continued elongating, more secondary and tertiary branches were formed and the inflorescence was nearly coming to anthesis stage (Fig. 1, f).

**Fig. 2:**
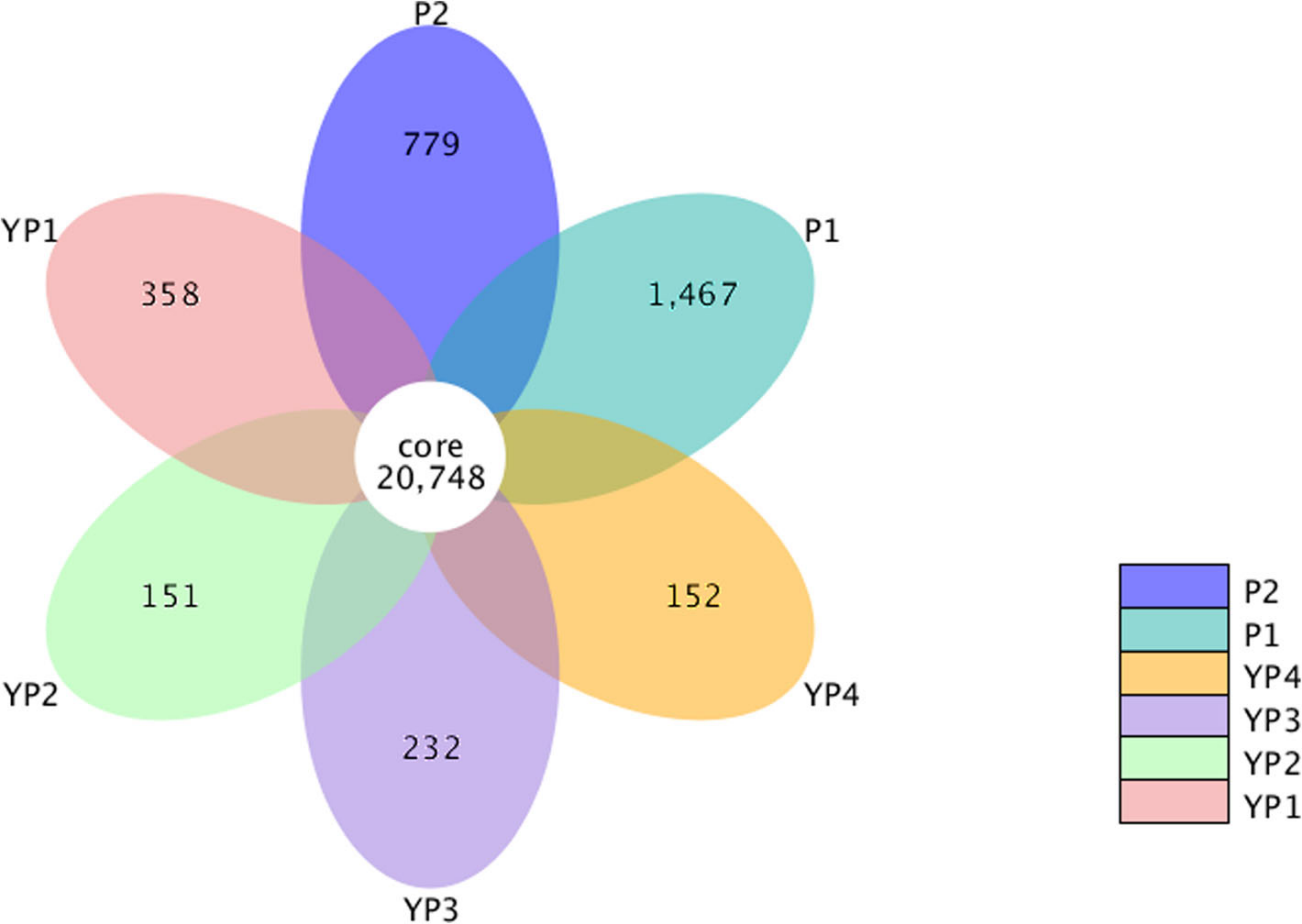
Venn diagram demonstrating the expressed genes among different-stage inflorescences. The number in core circle means the number of genes expressed at all stage with a cutoff of FPKM = 1. The numbers in different color ellipses means the preferentially expressed genes with their FKPM≥1 at one stage but FKPM< 1 at the other stages

Eighteen libraries were sequenced using Illumina Hiseq2500 platform, yielding an average of about 26 million of paired-end reads (2 × 150 bp) per library, with the total size amounting to about 138.8 Gb. The Q20 and Q30 values of each library were larger than 97 and 93% (Table 1), respectively. Subsequently, the clean reads were mapped to the quinoa genomic sequences [42] (NCBI, *Chenopodium quinoa*, ASM168347v1) using HISAT [47] program. Approximately 96% of the clean reads were mapped to the reference genome, with 91% mapped to exon regions and about 4–6% mapped to intergenic regions (Table 1). Meanwhile, the transcripts identified by StringTie based network flow algorithm [48] without annotation on the reference genome were designated as novel genes (Additional file 13: Dataset 2). The relative expression level of each gene was normalized with Fragments Per Kilobase of transcript sequence per Millions base pairs sequenced (FPKM) method [49]. Pearson correlation coefficient between three biological replicates of different stage tissues varied from 0.916 to 0.988, indicating the replicates were highly similar (Additional file 1: Figure S1). We defined a gene with the FPKM≥1 in at least one stage as an expressed one. Based on such criteria, we detected 23,951, 24,426, 24,794, 24,786 expressed genes in YP1, YP2, YP3 and YP4, respectively, while a higher number of expressed genes appeared in P1 (26,770) and P2 (25,592) (Additional file 3: Table S1). Among the expressed genes, 20,748 genes displayed common expression patterns with their FPKM≥1 at all the stages (Fig. 2). Then, we analyzed the transcriptome data to find out the genes conferred FPKM≥1 exclusively at one stage but FPKM< 1 at the other stages. Consequently, we found that 358, 151, 232 and 152 genes were preferentially expressed in YP1, YP2, YP3 and YP4, respectively, with P1 and P2 harboring relative higher numbers (1467 and 779) (Fig. 2), suggesting more distinct transcriptional activities occurred at P1 and P2 stages than at other stages.

**Table 1:** Summary of the RNA-seq data in this study yielded from Illumina Hiseq2500 platform

Sample	Raw reads (nt)	Clean reads (nt)	Clean data (Gb)	Q20 percentage	Q30 percentage	GC percentage	Total mapping rate	Exon mapping rate	Intron mapping rate	Intergenic mapping rate
YP1_1	25,695,596	25,307,873	7.59Gb	98.34%	94.97%	42.86%	96.86%	92.52%	3.04%	4.44%
YP1_2	23,961,907	23,550,226	7.07Gb	97.64%	93.25%	42.59%	96.28%	91.83%	3.50%	4.67%
YP1_3	24,090,997	23,756,749	7.13Gb	98.29%	94.86%	42.99%	96.91%	92.81%	2.63%	4.57%
YP2_1	22,458,101	22,064,401	6.62Gb	98.17%	94.55%	42.76%	96.62%	91.86%	3.51%	4.63%
YP2_2	26,322,924	25,933,325	7.78Gb	98.35%	94.97%	42.59%	96.82%	92.08%	3.38%	4.54%
YP2_3	28,597,224	28,207,492	8.46Gb	98.41%	95.20%	42.69%	96.79%	92.28%	3.20%	4.52%
YP3_1	31,454,462	30,971,613	9.29Gb	98.38%	95.09%	42.53%	96.88%	91.66%	3.60%	4.74%
YP3_2	24,607,612	24,122,774	7.24Gb	98.40%	95.15%	42.66%	96.74%	92.41%	3.38%	4.21%
YP3_3	28,119,471	27,654,238	8.30Gb	98.30%	94.91%	42.64%	96.8%	91.74%	3.51%	4.75%
YP4_1	26,766,950	26,483,040	7.94Gb	97.99%	93.86%	42.82%	96.88%	92.44%	2.94%	4.62%
YP4_2	27,739,079	27,271,759	8.18Gb	98.21%	94.65%	42.55%	96.5%	91.63%	3.75%	4.62%
YP4_3	27,486,903	27,022,147	8.11Gb	98.34%	94.99%	42.80%	96.83%	92.15%	3.38%	4.47%
P1_1	24,810,744	24,372,086	7.31Gb	98.22%	94.70%	42.69%	96.67%	91.33%	3.55%	5.11%
P1_2	26,317,484	25,820,278	7.75Gb	98.34%	95.01%	42.15%	96.9%	91.97%	1.87%	6.17%
P1_3	25,737,276	25,343,570	7.60Gb	98.08%	94.30%	42.69%	96.61%	91.70%	3.34%	4.96%
P2_1	25,958,754	25,582,291	7.67Gb	98.20%	94.68%	42.67%	96.24%	91.62%	3.48%	4.90%
P2_2	25,124,353	24,744,388	7.42Gb	98.30%	94.90%	42.87%	96.89%	92.12%	3.23%	4.65%
P2_3	25,010,070	24,633,309	7.39Gb	98.09%	94.39%	42.77%	96.75%	91.87%	2.95%	5.19%

### Differentially expressed genes at different stages

**Fig. 3:**
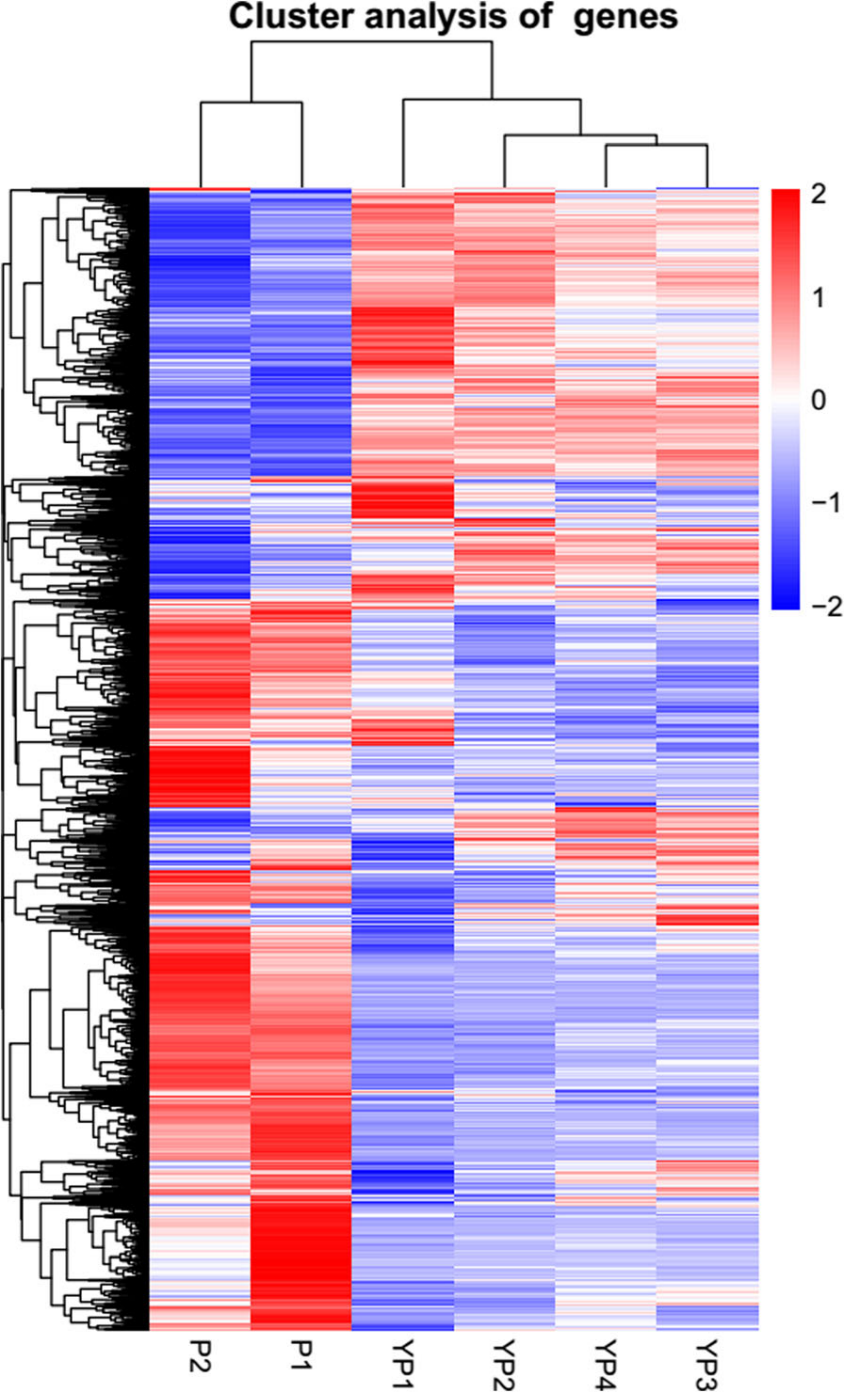
Heat map showing clustering of DEGs identified among different inflorescence tissues. Clustering was performed based on the normalized log10 (FPKM+ 1) value of each gene throughout the whole stages. Clearly, the DEGs expression profiles indicate the six tissues could be grouped into two major clades, one containing YP1, YP2, YP3 and YP4 and the other one containing P1 and P2. Red and Blue short lines stand for relatively higher and lower expression levels, respectively

Differential expression pattern analysis was performed using DESeq2 [50] R package (1.16.1) with the threshold of adjusted *P* value< 0.05. Through pairwise comparison, in total we identified 21,610 DEGs (Additional file 4: Table S2). Clustering analysis of these DEGs based on the FPKM values demonstrated that gene expression models in YP1 to YP4 inflorescence tissues were categorized into one major clade, which was distinguished from another clade consisting of P1 and P2 samples (Fig. 3). The numbers of DEGs between different groups varied from 145 to 13,356, where comparison between P2 and YP1 yielded the highest whereas that between YP4 and YP3 yielded the lowest number (Additional file 4: Table S2). Moreover, we observed that more temporally separated inflorescences samples yielded more DEGs, suggesting the transcriptional events change dramatically as development duration increased. We inspected the gene expression levels at different continuous intervals, and found comparisons between YP2 and YP1, P1 and YP4 generated more DEGs (2441 for YP2 vs YP1, 8335 for P1 vs YP4) than that in other comparisons (691 for YP3 vs YP2, 145 for YP4 vs YP3, 649 for P2 vs P1) (Additional file 4: Table S2), indicating more variation in gene expression at the early and late reproductive periods.

Furthermore, we performed GO and KEGG assignments to clarify the potential functions of DEGs at different intervals. According to the GO enrichment results, the up-regulated genes in YP2-vs-YP1 comparison were overrepresented in eleven GO items, including “carbohydrate biosynthetic process” and “protein heterodimerization activity” (Additional file 5: Table S3). In contrast, the down-regulated genes were significantly enriched in sixty-six items, such as “photosynthesis”, “translation”, “photosynthetic membrane” and “structural constituent of ribosome” (Additional file 5: Table S3). For YP3-vs-YP2 comparison, nineteen GO terms showed highly enrichment, in which “amine metabolic process” and “nucleic acid binding transcription factor activity”, and “ribosome” and “protein heterodimerization activity” were highly enriched in up- and down-regulated DEGs (Additional file 5: Table S3), respectively. During YP3 to YP4 phase, thirty-seven terms, such as “cellular carbohydrate metabolic process”, “extracellular region”, “xyloglucan:xyloglucosyl transferase activity” and “nucleic acid binding transcription factor activity” were enriched in up-regulated DEGs, whereas twenty-five terms containing “cellular component biogenesis” and “nucleosome” significantly appeared in down-regulated DEGs (Additional file 5: Table S3). Classification of DEGs in P1-vs-YP4 comparison yielded the largest number of GO items. The up-regulated genes were abundant in ninety-two GO terms including “cellular carbohydrate metabolic process”, “apoplast” and “oxidoreductase activity, and acting on peroxide as acceptor” (Additional file 5: Table S3). Meanwhile, the down-regulated genes were enriched in forty GO terms which encompassed “DNA replication”, “chromosome” and “microtubule motor activity” (Additional file 5: Table S3). For P2-vs-P1, the terms “response to oxidative stress”, “apoplast” and “peroxidase activity” were highly represented in up-regulated genes while “trehalose biosynthetic process” and “phosphoric ester hydrolase activity” were enriched in down-regulated genes (Additional file 5: Table S3).

The KEGG enrichment analysis results showed that at early stage (in YP2-vs-YP1), a large proportion of up-regulated DEGs were enriched in pathways “Plant hormone signal transduction” and “Biosynthesis of secondary metabolites”, and on the other hand “Photosynthesis-antenna proteins”, “Ribosome” were overrepresented in down-regulated DEGs (Table 2). The DEGs in YP3-vs-YP2 and YP4-vs-YP3 comparisons were enriched in pathways “Diterpenoid biosynthesis”, “Flavonoid biosynthesis” and “Ribosome” (Table 2). At late reproductive stage (in P1-vs-YP4), twenty pathways were enriched, out of which “Carbon fixation in photosynthetic organisms”, “Phenylpropanoid biosynthesis”, “Carbon metabolism”, “Nitrogen metabolism” as well as “Diterpenoid biosynthesis” and “Flavonoid biosynthesis” were overrepresented in DEGs with increased expression levels, while “DNA replication” was highly enriched in DEGs with decreased expression levels (Table 2). In P2-vs-P1, we found “Fatty acid elongation” and “Starch and sucrose metabolism” were significantly related to the up- and down-regulated DEGs (Table 2), respectively. The GO and KEGG enrichment results could provide useful cues for deciphering the genes involved in quinoa inflorescence morphogenesis.

**Table 2:** KEGG classification of the DEGs at different intervals

	Up-regulated		Down-regulated	
	Item	*p*-value	Item	*p*-value
YP2-vs-YP1	Plant hormone signal transduction	0.030906063	Photosynthesis - antenna proteins	7.75E-08
	Biosynthesis of secondary metabolites	0.035863996	Ribosome	4.67E-06
			Porphyrin and chlorophyll metabolism	2.14E-05
			Photosynthesis	2.14E-05
			Carbon fixation in photosynthetic organisms	0.0094376
			Nitrogen metabolism	0.0207042
			Arginine biosynthesis	0.0207042
YP3-vs-YP2	Diterpenoid biosynthesis	0.005119967	Ribosome	1.92E-13
YP4-vs-YP3	Flavonoid biosynthesis	0.025536677	Ribosome	0.0362887
	Nitrogen metabolism	0.025536677	Sulfur metabolism	0.0362887
	Ubiquinone and other terpenoid-quinone biosynthesis	0.025536677		
	Phenylalanine metabolism	0.025536677		
	Phenylpropanoid biosynthesis	0.049009994		
P1-vs-YP4	Carbon fixation in photosynthetic organisms	0.000192461	DNA replication	2.28E-05
	Phenylpropanoid biosynthesis	0.000192461	Mismatch repair	0.0059843
	Carbon metabolism	0.000818142	Purine metabolism	0.0190094
	Nitrogen metabolism	0.001083904	Pyrimidine metabolism	0.0194897
	Glyoxylate and dicarboxylate metabolism	0.003257291	Homologous recombination	0.0215846
	Starch and sucrose metabolism	0.00366431		
	Plant-pathogen interaction	0.004811801		
	Photosynthesis - antenna proteins	0.005158419		
	Alanine, aspartate and glutamate metabolism	0.016187972		
	Photosynthesis	0.020905938		
	Diterpenoid biosynthesis	0.026926862		
	Flavonoid biosynthesis	0.034576313		
	Pentose and glucuronate interconversions	0.034576313		
	Phenylalanine metabolism	0.04782332		
	Fructose and mannose metabolism	0.04782332		
P2-vs-P1	Fatty acid elongation	0.048695132	Starch and sucrose metabolism	0.0019501
			Galactose metabolism	0.048367

DEGs were subjected to KEGG enrichment analysis using cluster Profiler R package. Items with *P* value less than 0.05 were considered significantly enriched

### Transcription factor dynamics during inflorescence development

**Fig. 4:**
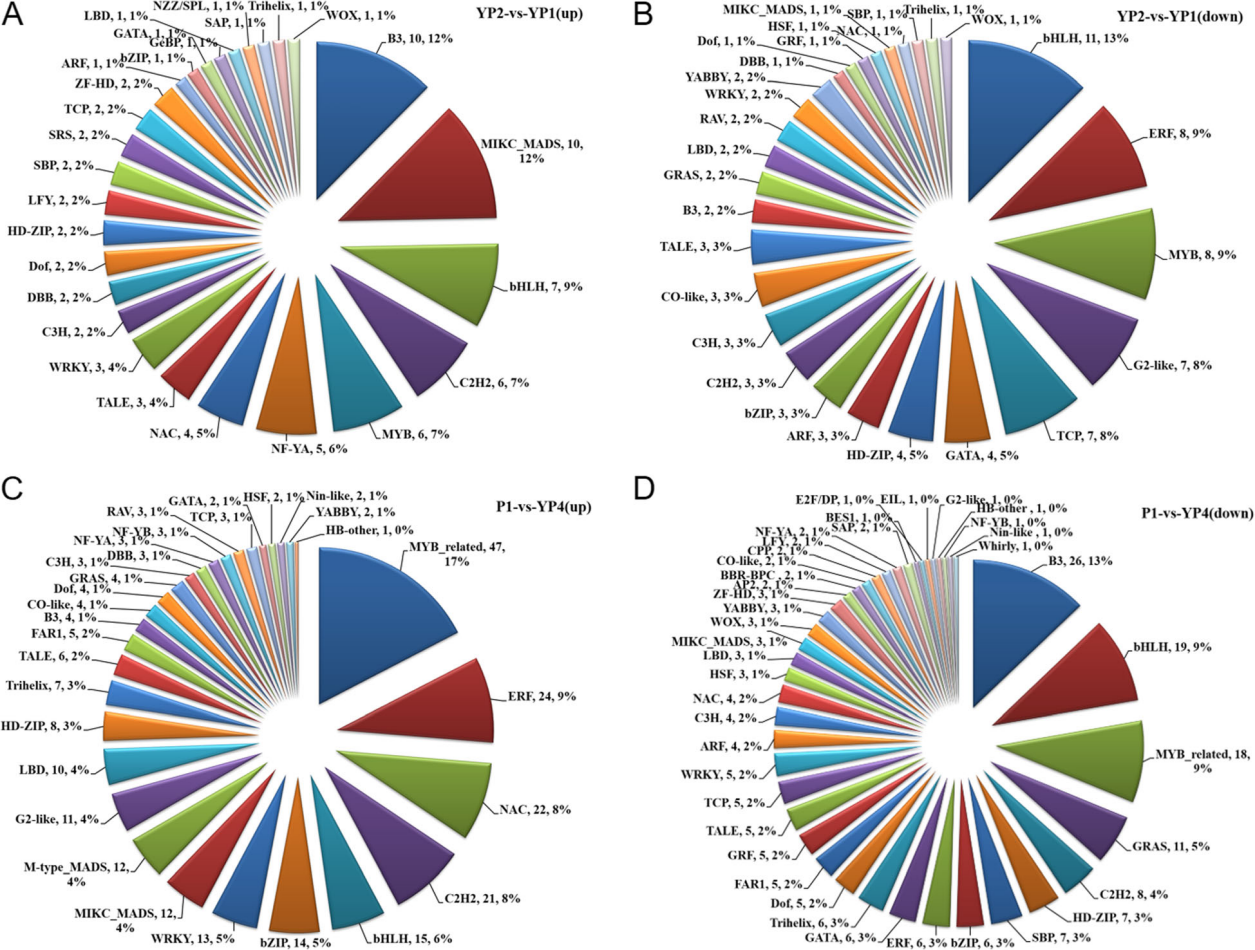
Composition of the transcription factors in up- or down-regulated DEGs at early and late reproductive stages. The transcription factor (TF) constitution of early interval (YP2-vs-YP1) (**a**, **b**) varies from that of late interval (P1-vs-YP4) (**c**, **d**). Each sector represents one TF family. The number after each TF name represents the TF number and its proportion

Transcription factors (TFs) are regarded as critical regulators in many plant developmental events. In order to survey the TFs dynamics during all quinoa inflorescence stages, we identified the genome-wide TF encoding genes and the TFs in the DEGs using an online TF prediction tool in Plant Transcription Factor Database v4.0 [51]. As illustrated in Additional file 6: Table S4, in up- or down-regulated DEGs at different periods TFs accounted for considerable proportion (approximately 4.26 to 16.67%) (Additional file 2: Figure S2, A), which was larger than the genome-wide TFs proportion (4.14%) (Additional file 7: Table S5). Among all developmental intervals, the phases YP2-vs-YP1 and P1-vs-YP4 have the highest numbers of differentially expressed TFs (Additional file 6: Table S4). One hundred sixty nine TF encoding genes out of 2441 DEGs (accounting for 6.9%) were identified in YP2-vs-YP1 comparison (Additional file 6: Table S4). By comparing the TF proportion in DEGs with that in the genome background (Additional file 2: Figure S2, B), we found B3, MIKC-type MADS-box and C2H2 family members were highly more enriched in the up-regulated TFs whereas bHLH, ERF (Ethylene-Response Factor), MYB, TCP (TEOSINTE BRANCHED, CYCLOIDEA and PCF), G2-like, GATA and HD-ZIP family members were significantly more abundant in down-regulated TFs (Fig. 4, a-b). In P1-vs-YP4 comparison, 270 and 202 TFs encoding genes represented in 5841 up- and 2494 down-regulated DEGs (Additional file 6: Table S4), respectively. In the same way, ERF, NAC, C2H2, bZIP, WRKY, MADS-box (including MIKC-type and M-type MADS), G2-like and LBD encoding genes were highly more enriched in up-regulated TFs (Fig. 4c). In contrast, B3, bHLH, GRAS, HD-ZIP, SBP and GATA members were more abandunt in down-regulated DEGs (Fig. 4d). Interestingly, MYB was overrepresented in up-regulated as well as in down-regulated DEGs (Fig. 4, c-d). These TFs mentioned above were also identified in YP3-vs-YP2, YP4-vs-YP3 and P2-vs-P1.

In addition, we found several important inflorescence development related TFs expressed in all stages, including LFY, CO-like (CONSTANS-like), WOX (WUSCHEL-related homeobox), TALE, AP2, DOF and YABBY (Additional file 6: Table S4; Fig. 4, a-d). The TFs profiling at different intervals could supply with cues for further investigation of the inflorescence morphology changes.

### Weighted gene co-expression network analysis of DEGs

**Fig. 5:**
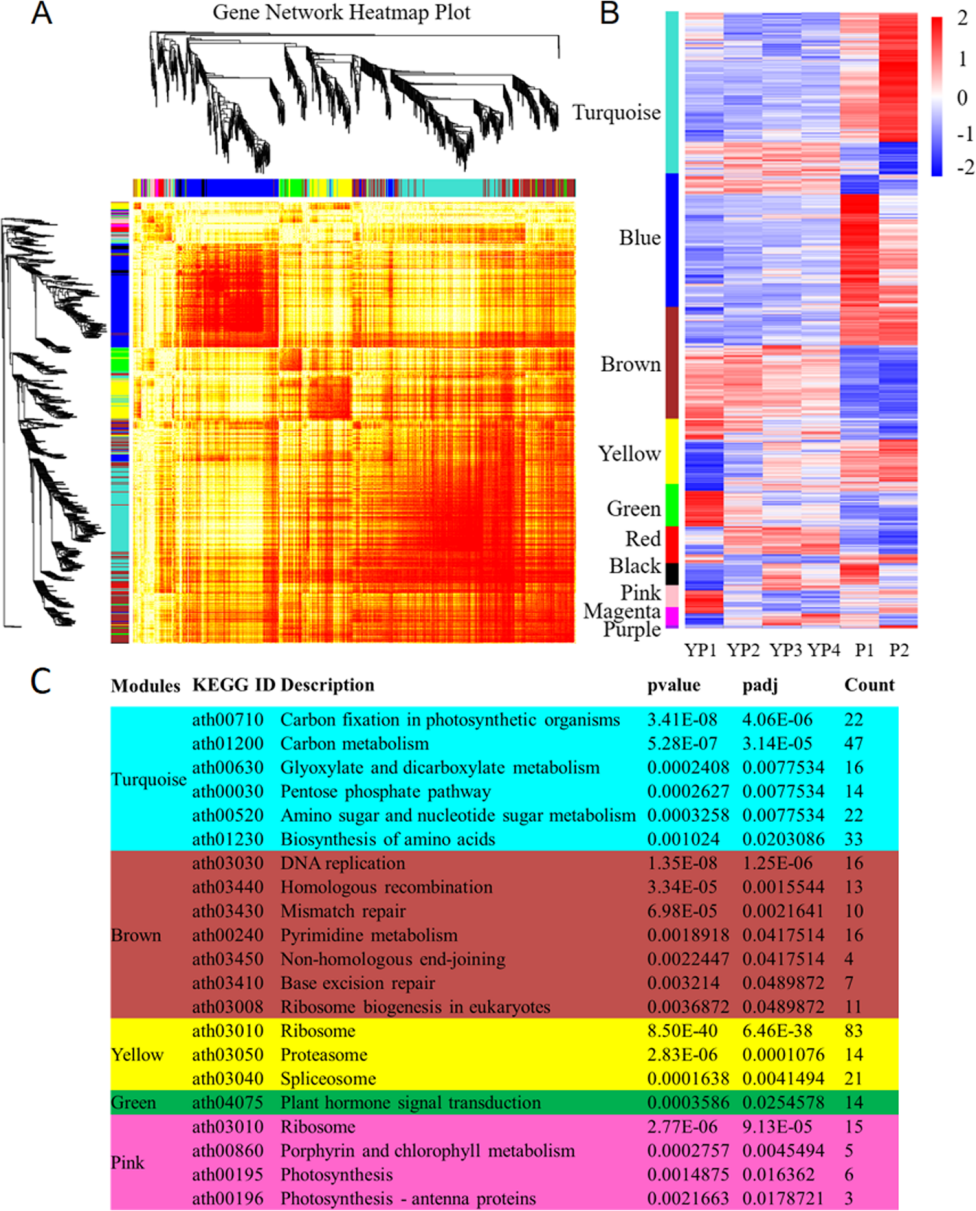
Weighted gene co-expression network analysis of DEGs. **a** 21,568 out of 21,610 DEGs are grouped into ten gene modules. 5949, 5410, 4442, 2206, 1532, 836, 417, 404, 340 and 32 DEGs were co-expressed in the Turquoise, Blue, Brown, Yellow, Green, Red, Black, Pink, Magenta and Purple modules, respectively. **b** Heat map showing the gene expression levels of DEGs in different modules. **c** KEGG enrichment analysis of different gene sets

Weighted Gene Co-Expression Network Analysis (WGCNA) [52] is a powerful tool for generating gene co-expression networks for RNA-seq analysis. Using this tool co-regulated genes that may possess similar biological function could be identified. To uncover the potential regulatory networks regarding to quinoa inflorescence development, we carried out WGCNA on the DEGs. Totally, we found 21,568 out of 21,610 DEGs (99.8%) across all the inflorescence developmental stages could be categorized into 10 modules (Fig. 5a), with the Turquoise module harboring the most genes (5949 DEGs) whereas the Purple module harboring the least genes (32 DEGs) (Additional file 8: Table S6). Remarkably, the majority of the genes in the Turquoise, Blue, Green, Black, Pink and Purple modules showed highly specific expression pattern at one single stage. As showed in Fig. 5b, the Turquoise and Purple modules were significantly more abundant at P2 stage, the Blue and Black modules were mainly expressed at P1 stage, whereas the Green and Pink modules showed high expression at YP1 stage (Fig. 5b).

To further investigate the biological functions of these gene modules, we performed KEGG analysis. As detailed in Fig. 5c, for the Turquoise module, pathways including “Carbon fixation in photosynthetic organisms”, “Carbon metabolism”, “Glyoxylate and dicarboxylate metabolism”, “Pentose phosphate pathway”, “Amino sugar and nucleotide sugar metabolism” and “Biosynthesis of amino acids” were overrepresented (Fig. 5c). The Brown module was abundant in “DNA replication”, “Homologous recombination”, “Mismatch repair”, “Pyrimidine metabolism”, “Non-homologous end-joining”, “Base excision repair” and “Ribosome biogenesis in eukaryotes” (Fig. 5c). The Yellow module contained most genes enriched in “Ribosome”, “Proteasome” and “Spliceosome”. The Green module was only highly enriched in “Plant hormone signal transduction” (Fig. 5c). For the Pink module, “Ribosome”, “Porphyrin and chlorophyll metabolism”, “Photosynthesis” and “Photosynthesis-antenna proteins” were significantly enriched (Fig. 5c).

### Identification of the core transcriptional regulators modulating inflorescence architecture

Gene sets that show highly stage-specific expression patterns usually are closely related to the corresponding phenotypes. To investigate the regulatory networks shaping inflorescence architecture, we intensively focused on the modules including Green and Pink with high specific expression at early reproductive phase panicles (none branching stage), and the modules including Turquoise, Blue, Black, and Purple with high specific expression at late reproductive phase panicles (branching stage). As TFs play essential roles for the morphogenesis of various plant tissues and organs, we sorted out the TF encoding genes of each module. Further, we generated core transcriptional regulatory networks of each module using the TFs (Additional file 9: Table S7) and key homologs (Additional file 10: Table S8**)** involved in inflorescence architecture regulation as seed nodes.

**Fig. 6:**
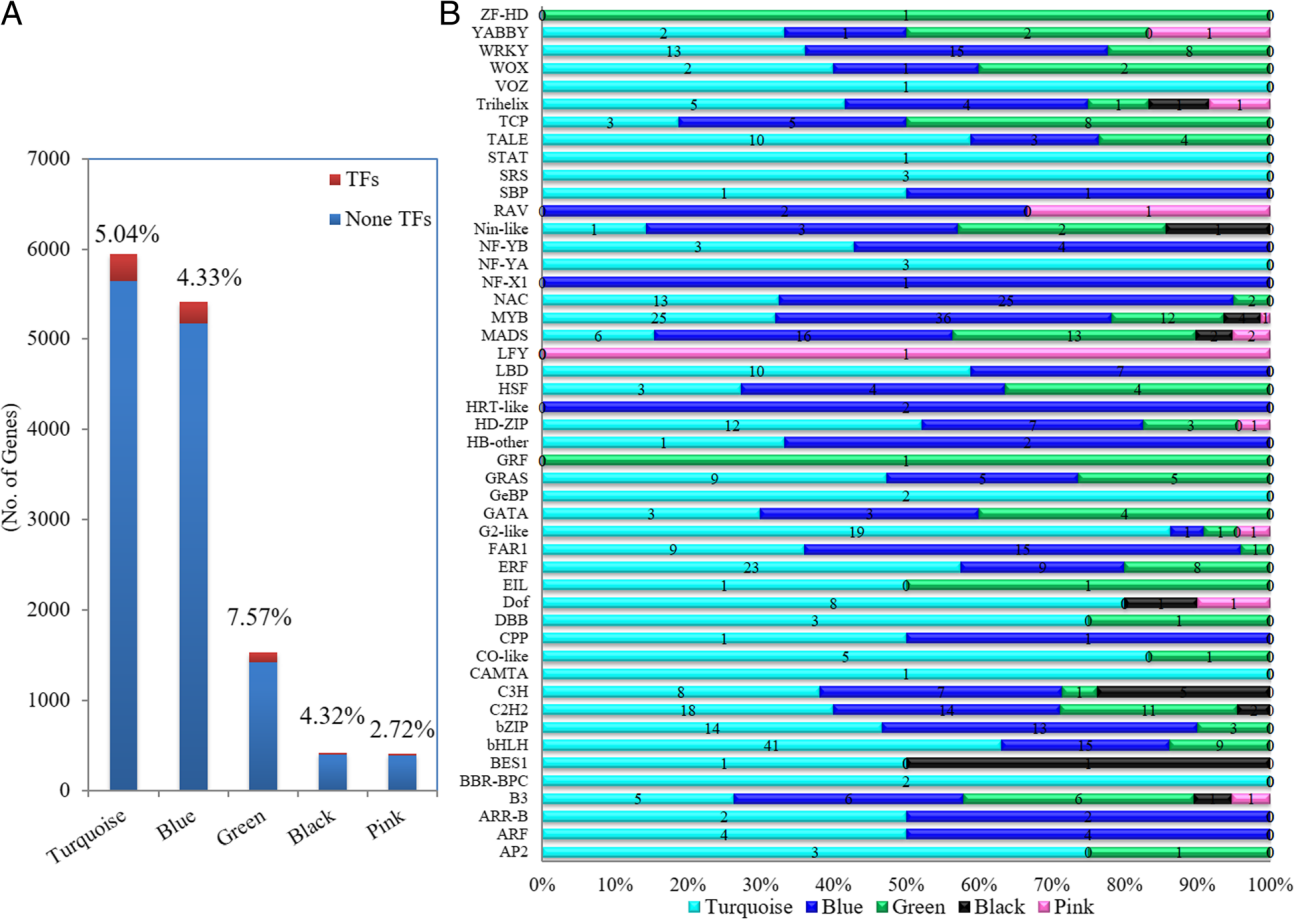
Distribution of transcription factors in Turquoise, Blue, Green, Black and Pink module. **a** Statistic result of the TFs number and its proportion at each gene module. **b** the composition of TF family members distributed at different gene modules

Totally, with the exception that Purple harbored none TFs, 48 different TF families were identified among these modules (Additional file 9: Table S7). The Turquoise module harbored the largest TF number (300), and the Green module harbored the highest TF proportion (7.57%) (Additional file 9: Table S7, Fig. 6a). Obviously, the TF percentages of these modules were larger than that in the genome background with the exception of the Pink module (2.72% vs 4.14%) (Fig. 6a). Notably, some TFs were only present in one module, such as LFY (Gene ID: 110698679) in the Pink module, ZF-HD (Gene ID: 110717138) and GRF (Gene ID: 110738890) in the Green module, and VOZ (Gene ID: 110693688), STAT (Gene ID: 110694069), SRS (Gene ID: 110723239, 110,724,730, novel.32919), NF-YA (Gene ID: 110684358, 110,737,411, novel.26441), GeBP (Gene ID: 110687270, 110,693,452), CAMTA (Gene ID: 110697210) and BBR-BPC (Gene ID: 110686586, 110,692,413) in the Turquoise module (Fig. 6b). Further, we found the TF composition varied among different modules. For instance, bHLH is the largest TF family in the Turquoise module; the Blue module harbors the most MYB members; MADS tops in the Green and Pink modules; while C3H tops in the Black module (Fig. 6b). The module-specific TF composition may render special effects for inflorescence development.

**Fig. 7:**
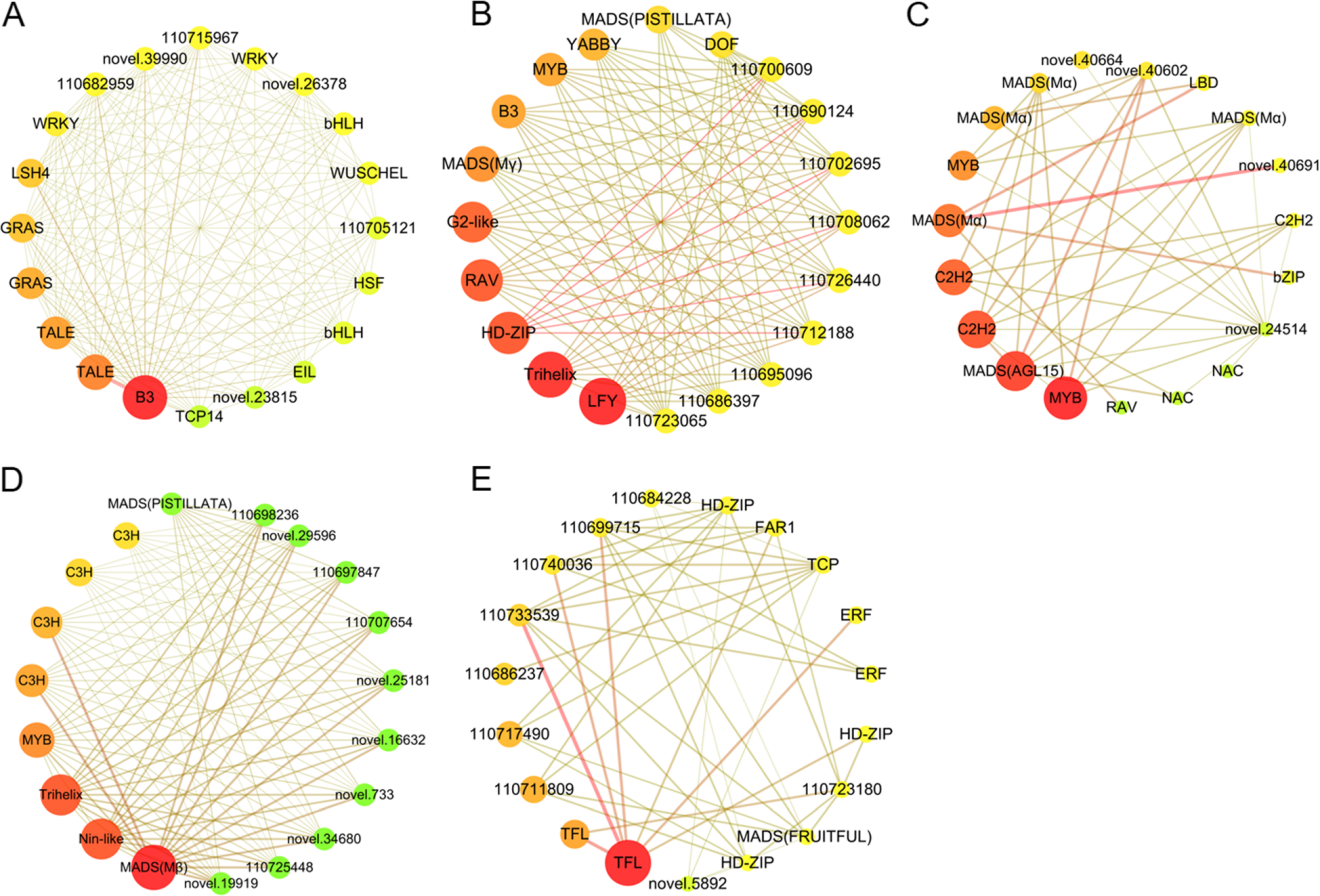
Central hubs of the core co-expression networks involved in maintaining young-status and mature-status inflorescence. The core regulatory network was generated from each module with stringent edge weight cutoffs by using the TFs and crucial plant inflorescence development related homologs as seed nodes. The nodes with top degrees were defined as central hubs in each core network. **a** for the Green module, a core network composed of 602 nodes and 2253 edges were generated using an edge weight cutoff of 0.50; (**b**) for the Pink module, a core network composed of 111 nodes and 394 edges was generated using an edge weight cutoff of 0.30; (**c**) for the Blue module, a core network composed of 649 nodes and 2055 edges was generated using an edge weight cutoff of 0.58; (**d**) for the Black module, a core network composed of 227 nodes and 1021 edges was generated using an edge weight cutoff of 0.55; (**e**) for the Turquoise module, a core network composed of 232 nodes and 499 edges was generated using an edge weight cutoff of 0.58. The networks were visualized using Cytoscape. The nodes with larger size and darker red indicate greater connectivity within the network

In order to screen the principle regulators participating in maintaining inflorescence morphology at different stages, we manually refined the corresponding modules with proper edge weight cutoffs and visualized each core networks using Cytoscape [53]. As detailed in Table 3, we found the central hubs in modules related to early reproductive phase inflorescences significantly varied from that in modules related to late reproductive phase inflorescences. For the Green module, a core network containing 602 nodes and 2253 edges were obtained. Using Cytoscape we filtered to obtain the largest 20 hubs with top degrees, among which 14 were TF encoding genes (Fig. 7a, Table 3). Specifically, the largest hub 110,723,560 is a B3 TF. 110,692,073 encodes a WUSCHEL-related homeobox (WOX) protein which is important for floral meristem development in *Arabidopsis* [16] (Fig. 7a). 110,715,387 and 110,705,456 are TALE family members that share homeodomain with KNOX and WOX proteins. 110,717,078 and 110,682,413 encode GRAS TFs resembling the rice MONOCULM 1 (MOC1, [54]) that controls tiller number and panicle branches. 110,708,326 encodes a LSH protein belonging to the same ALOG family with spikelet number regulator TAW1 [35]. 110,700,571 encodes a plant-specific TCP, resembling the maize TB [55], the rice OsTB1 and the homologous BRANCHED1 (BRC1) proteins in *Arabidopsis* [56], pea and tomato, which have conserved roles in suppressing branching. Besides, the core hubs also contained *WRKY* (Gene ID: 110710092 and 110,682,067), *bHLH* (Gene ID: 110714201 and 110,703,894), *HSF* (Gene ID: 110686357) and *EIL* (Gene ID: 110695891) (Fig. 7a). In another early reproductive phase related Pink module, we found 11 central hub genes were TFs in the refined core network (Fig. 7b). Especially, the largest hub encodes a LFY protein (110698679) (Fig. 7b) whose homologs have been widely identified as crucial floral identity genes in many other plant species [3, 57]. The second largest hub is 110,729,369, encoding a Trihelix protein. 110,736,045 and novel.22515 encode an M-type (Mγ) and MICK-type (PISTILLATA-like) MADS-box protein, respectively, which are crucial for floral development [58]. Meanwhile, several plant development related genes, such as *DOF* (Gene ID: 110711979), *G2*-like (Gene ID: 110691257), *HD-ZIP* (Gene ID: 110729692) and *RAV* (Gene ID: 110719982), *B3* (Gene ID: novel.9281), *MYB* (Gene ID: 110708638) and *YABBY* (Gene ID: 110685646) were also identified as hub genes in this module (Fig. 7b).

**Table 3:** List of transcription factors with top degrees in the core network of each module

Module	Gene ID	Gene name	Degree	Best hit in A.thaliana	Description for the best hit
Green	110,723,560	B3	586	AT5G42700.1	B3 family protein
	110,715,387	TALE	355	AT4G32980.1	homeobox gene 1
	110,705,456	TALE	283	AT4G32980.1	homeobox gene 1
	110,717,078	GRAS	245	AT3G49950.1	GRAS family protein
	110,682,413	GRAS	184	AT3G49950.1	GRAS family protein
	110,708,326	LSH4	179	AT3G23290.2	LIGHT-DEPENDENT SHORT HYPOCOTYLS-like protein
	110,710,092	WRKY	76	AT5G43290.1	WRKY DNA-binding protein 49
	110,682,067	WRKY	36	AT4G39410.1	WRKY DNA-binding protein 13
	110,714,201	bHLH	33	AT4G37850.1	bHLH family protein
	110,692,073	WOX	29	AT3G18010.1	WUSCHEL related homeobox 1
	110,703,894	bHLH	27	AT3G26744.1	bHLH family protein
	110,686,357	HSF	27	AT1G46264.1	heat shock transcription factor B4
	110,695,891	EIL	25	AT5G65100.1	EIL family protein
	110,700,571	TCP	22	AT3G47620.1	TEOSINTE BRANCHED, cycloidea and PCF (TCP) 14
Pink	110,698,679	LFY	57	AT5G61850.1	floral meristem identity control protein LEAFY (LFY)
	110,729,369	Trihelix	55	AT5G03680.1	Trihelix family protein
	110,729,692	HD-ZIP	47	AT4G00730.1	HD-ZIP family protein
	110,719,982	RAV	46	AT1G13260.1	related to ABI3/VP1 1
	110,691,257	G2-like	44	AT1G25550.1	G2-like family protein
	110,736,045	M-type_MADS	33	AT5G48670.1	AGAMOUS-like 80
	novel.9281	B3	29	AT4G33280.1	B3 family protein
	110,708,638	MYB_related	28	AT3G21430.2	DNA binding
	110,685,646	YABBY	25	AT1G69180.1	YABBY family protein
	novel.22515	MIKC_MADS	16	AT5G20240.1	MIKC_MADS family protein
	110,711,979	Dof	14	AT2G28510.1	Dof family protein
Blue	novel.11405	MYB_related	316	AT4G09450.1	MYB_related family protein
	110,707,171	MIKC_MADS	282	AT5G13790.1	AGAMOUS-like 15
	110,688,475	C2H2	258	AT5G59820.1	C2H2 family protein
	110,684,112	C2H2	237	AT5G59820.1	C2H2 family protein
	110,685,095	M-type_MADS	214	AT5G60440.1	AGAMOUS-like 62
	novel.23507	MYB_related	176	AT5G04760.1	MYB family protein
	110,735,861	M-type_MADS	119	AT5G60440.1	AGAMOUS-like 62
	110,740,130	M-type_MADS	75	AT2G24840.1	AGAMOUS-like 61
	110,696,735	LBD	36	AT3G49940.1	LOB domain-containing protein 38
	110,736,081	M-type_MADS	34	AT5G60440.1	AGAMOUS-like 62
	110,737,201	C2H2	25	AT5G56200.1	C2H2 family protein
	110,697,372	bZIP	19	AT3G62420.1	basic region/leucine zipper motif 53
	110,726,622	NAC	15	AT3G04070.1	NAC domain containing protein 47
	110,726,634	NAC	15	AT3G04070.1	NAC domain containing protein 47
	110,719,209	RAV	14	AT1G50680.1	RAV family protein
Black	novel.4708	M-type_MADS	223	AT5G55690.1	M-type_MADS family protein
	novel.40662	Nin-like	181	AT2G43500.1	Nin-like family protein
	110,711,872	Trihelix	180	AT2G33550.1	Trihelix family protein
	110,687,197	MYB_related	128	AT5G04760.1	MYB family protein
	novel.34399	C3H	106	AT3G19360.1	C3H family protein
	110,698,972	C3H	93	AT3G19360.1	C3H family protein
	110,736,053	C3H	58	AT1G03790.1	C3H family protein
	novel.34398	C3H	50	AT3G19360.1	C3H family protein
	110,725,731	MIKC_MADS	10	AT5G20240.1	MIKC_MADS family protein
Turquoise	110,697,084	TFL	85	AT5G03840.1	TERMINAL FLOWER 1
	110,729,689	TFL	44	AT5G03840.1	TERMINAL FLOWER 1
	110,722,691	FAR1	18	AT1G10240.1	FAR1-related sequence 11
	110,722,837	HD-ZIP	18	AT5G47370.1	Homeobox-leucine zipper protein 4 (HB-4) / HD-ZIP protein
	110,694,654	TCP	18	AT3G27010.1	TCP family protein
	110,733,648	ERF	17	AT5G25190.1	ERF family protein
	110,711,921	ERF	16	AT5G13330.1	related to AP2 6 l
	110,722,774	HD-ZIP	15	AT3G61890.1	homeobox 12
	110,731,701	HD-ZIP	14	AT2G46680.1	homeobox 7
	110,722,241	MIKC_MADS	14	AT5G60910.1	AGAMOUS-like 8

The detailed information of the central TF hubs in Fig.7 is displayed in this table. The homologs of these hubs were identified in model plant *Arabidopsis thaliana* based on the prediction results obtained in Plant Transcription Factor Database v4.0 (http://planttfdb.cbi.pku.edu.cn)

Subsequently, we analyzed the modules closely related to late reproductive phase panicles. In the Blue module, the largest hub is a *MYB* gene (Gene ID: novel.11405) (Fig. 7c). Surprisingly, 5 out of 20 hubs are revealed to be MADS-box genes including one MICK-type (*AGL18-like*) (Gene ID: 110707171) and four M-type (*Mα*) (Gene ID: 110685095, 110,735,861, 110,740,130, 110,736,081) (Fig. 7c). Besides, we found co-regulated genes including *C2H2* (Gene ID: 110688475, 110,684,112, 110,737,201), *LBD* (Gene ID: 110696735), *bZIP* (Gene ID: 110697372) and *NAC* (Gene ID: 110726622 and 110,726,634) are specifically present as hubs in the Blue module rather than in other modules (Fig. 7c). In the Black module, the M-type (*Mβ*) MADS-box gene (Gene ID: novel.4708) harbors the most edges (Fig. 7d). Another MADS-box gene (Gene ID: 110725731), a homolog of the *Arabidopsis PISTILLATA* which is homeotic B-class gene involved in petal and stamen specification [58], was also present as a hub gene in this module (Fig. 7d). Interestingly, several *C3H* genes (Gene ID: novel.34399, 110,698,972, 110,736,053 and novel.34398) and a *Nin-like* (Gene ID: novel.40662) gene were identified as the specific central hubs in this module (Fig. 7d). In the core subnetwork of the Turquoise module, the largest two hubs 110,697,084 and 110,729,689 (Fig. 7e) were homologous to *TFL* which interacts with *AP1* and *LFY* to control floral transition as well as plant inflorescence architecture in many plants [3, 4]. 110,694,654 and 110,733,648 encode ERF TFs whose counterparts regulate floral meristem specification in *Arabidopsis* and rice. Remarkably, a MICK-type MADS-box encoding gene (Gene ID: 110722241), a homolog of the *Arabidopsis FRUITFULL* which controls meristem identity and fruit development [58], appeared as another hub in this module (Fig. 7e). Meanwhile, *HD-ZIP* (Gene ID: 110722837, 110,722,774, 110,731,701), *FAR1* (Gene ID: 110722691) as well as *TCP* (Gene ID: 110694654) genes were also included in this late reproductive stage associated module (Fig. 7e).

### Real-time validation of RNA-seq data

**Fig. 8:**
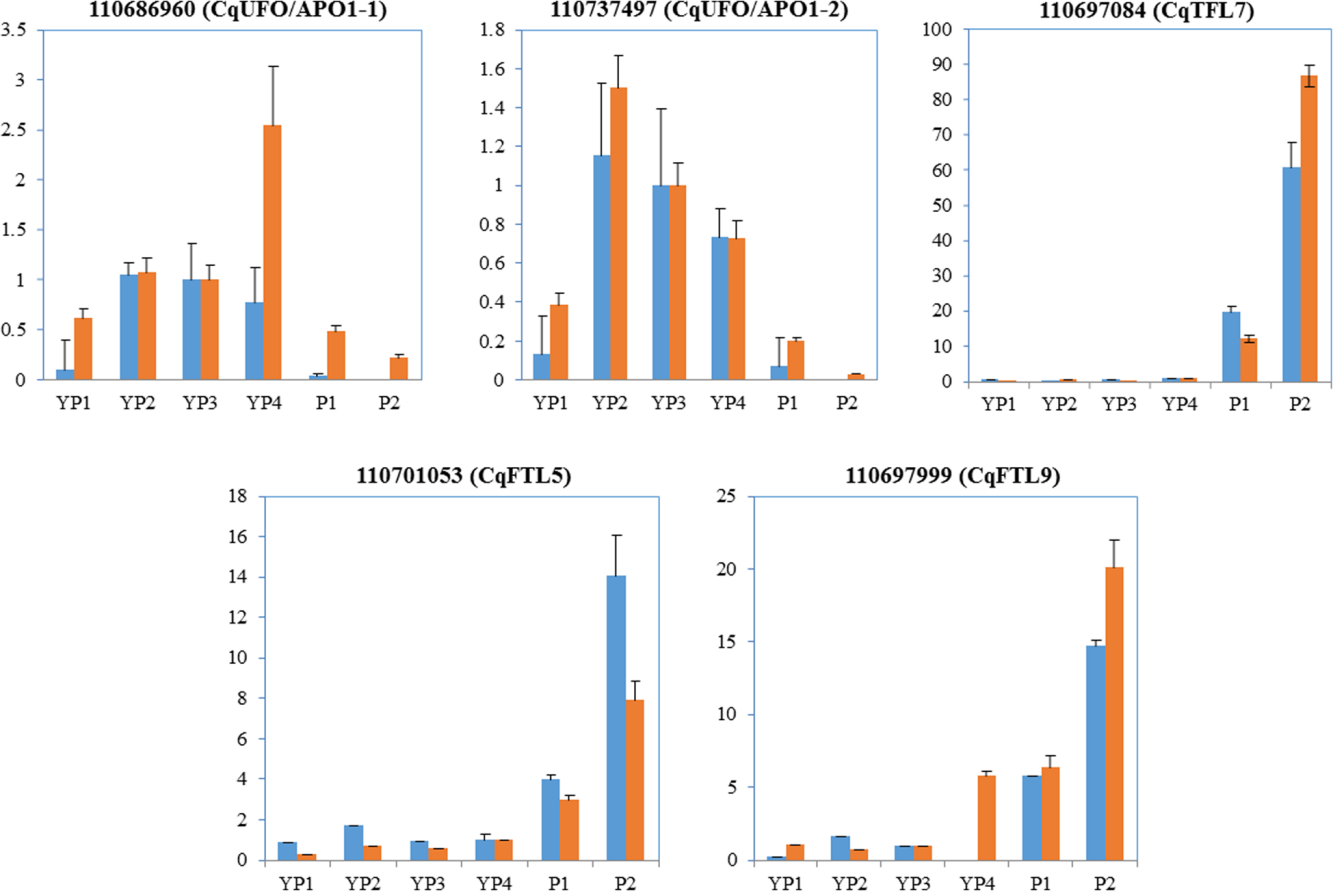
Real-time PCR validation of genes expression levels obtained by RNA-seq. The light blue and orange bars stand for relative gene expression levels obtained by RNA-seq and real-time PCR, respectively. The value of real-time PCR was calculated based on the 2^−ΔΔCt^ algorithm. Values are mean ± SD (*n* = 3)

To test the reliability of RNA-Seq data, we performed real-time PCR to measure the expression levels of five genes at six stages, including *CqAPO1–1* (Gene ID: 110686960), *CqAPO1–2* (Gene ID: 110737497), *CqTFL7* (Gene ID: 110697084), *CqFTL5* (Gene ID: 110701053) and *CqFTL9* (Gene ID: 110697999). Then we normalized and compared the real-time PCR results with that of RNA-Seq data. We found the gene expression levels measured by real-time PCR were highly consistent with that obtained from RNA-Seq analysis (Fig. 8), suggesting the high quality of the transcriptome data.

## Discussion

Improvement of inflorescence or panicle structure could effectively increase the grain yield and thus it is an important rational designing concept [5]. Over past decades, the key advances in the model plant *Arabidopsis*, in grasses as well as in legumes have shed lights on how the biological events including floral transition, panicle branching and spikelet formation interact with each other and contribute to the final yield. Though many regulators act in conserved manners among different plant species, however, divergent nodes still exist which may be responsible for the distinct inflorescence architectures [3]. Therefore, detailed investigation should be conducted especially for poorly understood plants. Quinoa is an important high-nutritional pseudo cereal, but maximizing its yield potential is still under progress to meet the worldwide demands [45]. In the present study, we conducted comprehensive investigation on quinoa inflorescence development, and generated regulatory networks patterning early reproductive phase and late reproductive phase inflorescence architecture using RNA-seq and Weighted Gene Co-Expression Network Analysis (WGCNA) methods.

We performed transcriptomic analysis using the quinoa inflorescence samples collected from six stages after floral transition. Pearson correlation analysis and clustering of the differentially expressed genes (DEGs) indicated the developmental activities in YP1, YP2, YP3 and YP4 samples resembled each other but were divergent from P1 and P2 samples. KEGG analysis of the DEGs at early reproductive intervals yield distinct pathway items compared with that yielded at late intervals. For example, the up- and down-regulated DEGs between YP2 and YP1 were enriched in “Plant hormone signal transduction” and “Photosynthesis”, respectively. By contrast, the up- and down-regulated DEGs between P1 and YP4 were overrepresented in “Carbon fixation in photosynthetic organisms” and “DNA replication”, respectively. These pathway differences were accompanied with transcriptional activities variation which was reflected by the transcription factors (TFs) composition of DEGs at different intervals. Thus, characterization of these different-stage transcriptomes could facilitate identifying the potential key regulators involved in inflorescence architecture formation. However, large-scale transcriptome analysis usually yields a great deal of DEGs, increasing the difficult in screening potential candidates. Besides, inflorescence development processes are usually controlled by a great complex of regulators, hence identification of the hub genes demands very effective analysis methods. WGCNA is a very powerful tool for identifying key regulators in big data analysis. Recently, its application has elucidated the complex networks of many important agronomic traits, such as wheat spike architecture [59], wild strawberry flower and fruit development [60], and chickpea seed development [61].

In this study, by using WGCNA, 21,610 DEGs identified across all developmental stages were divided into 10 modules. According to the expression profiles of these modules, we found gene sets in five modules representing early (YP1) and late induction (P1/P2) of gene expression. Expression levels of the Green and Pink modules peaked at young panicles (YP1), and the Turquoise, Blue, Black and Purple modules showed specific expression at late reproductive phase panicles (P1 and P2). As YP1 inflorescences showed no obvious branching phenotypes compared with P1 and P2 inflorescences, we designated that YP1 inflorescences were not branched whereas P1 and P2 inflorescences had experienced branching. Consequently, we mainly explored YP1, P1 and P2 associated modules. KEGG enrichment analysis of the early induced modules (Green/Pink) highlights the roles of “Plant hormone signal transduction”, “Ribosome” and “Photosynthesis” at YP1 stage, while the late induced module (Turquoise) is highly enriched in “Carbon fixation in photosynthetic organisms” and “Carbon metabolism”, suggesting phytohormone signaling is essential for inflorescence development before branching while carbon source energy is important for branching occurrence.

Transcriptional regulation has pivotal role in inflorescence development, up to date most of the unveiled inflorescence architecture regulators are TF encoding genes [4]. Therefore, in the present study we emphasized on investigating the TF associated subnetworks. Before construction of core networks, we manually refined the networks with stringent cutoffs to filter out edges with low edge weights. Then, we used the TFs and the key inflorescence-shaping related homologs as seed nodes to extract the coexpression networks. As hub genes are principle regulators in modules, we designated the 20 genes with top connectivity as central hubs in each network. Clearly, most hub components in early reproductive phase related modules are different from that in late reproductive phase related modules. For example, *B3*, *TALE*, *WOX*, *LSH*, *LFY*, *GRAS*, *bHLH*, *EIL*, *DOF*, *G2-like* and *YABBY* family members uniquely occurred in the Green and Pink modules, whereas *TFL*, *ERF*, *bZIP*, *HD-ZIP*, *C2H2*, *LBD*, *NAC*, *C3H*, *Nin-like* and *FAR1* specifically occurred in the Blue, Black and Turquoise modules as central hubs. Consistently, some of these hub homologs were deciphered as inflorescence controllers in previous cases. Mutation of the *TALE* family member *KNOX* and the *WOX* family member *WUS* renders smaller inflorescence and less branching morphologies [15, 18, 62]. Gain-of-function mutation in the *LSH* family member *TAW1* causes prolonged branching period and increased yield [35]. Overexpression of a rice *DOF* member-*OsDOF12* leads to reduced panicle size [63]. The *GRAS* family member *MOC1* is required for tiller and panicle branching in rice [54]. *LFY* functions as floral identity gene in *Arabidopsis* and its homolog *APO2*/*RFL* functions as flowering time controller and panicle architecture modulator in rice [34]. *TFL* antagonizes with *AP1* to control floral transition and inflorescence architecture [30]. The *ERF* family member *FZP*/*BFL1* inhibits axillary meristems and activates florets specification [37]. Maize *RAMOSA 1* (*RA1*, [64]) and *RA2* [65] are involved in inflorescence development, belonging to *C2H2* and *LBD* family, respectively. Several cases indicate that *C2H2* and *C3H* genes not only participate in controlling flowering time, but also are involved in regulating panicle structure in rice [66, 67]. Thus, we speculate that the unique distribution pattern of these TF nodes may account for the distinct developmental status of non-branching (YP1) and branched (P1 and P2) inflorescence. *MADS* genes have versatile roles in regulating flowering time, inflorescence architecture, floral specification, and fruit and seed development [58, 68]. In this case, we found *MADS* genes occurred in both none-branching and branched status samples, and the *MADS* nodes in branched inflorescences outnumbered that associated with none-branching inflorescences. Coincidently, recent transcriptome profiling studies also identified several *MADS* genes associated with spike architecture in wheat and barley (*Hordeum vulgare*) [69]. In our study, the identified *MADS* genes are inclusive of *Mα*, *Mβ*, *Mγ*, *PISTILLATA-like*, *FRUITFULL-like* and *AGL18-like* subfamilies. The detailed information regarding to the genetic roles of these *MADS* family members has been well reviewed [58, 68], indicating overlapping and divergent roles exist between different subfamilies. Therefore, we postulate that the different functions of these *MADS* members may be responsible for the inflorescence development and branching. Further functional characterization of these hub genes in the future will shed light on how they interact with each other to modulate quinoa inflorescence architecture.

## Conclusions

In the present study we carried out transcriptome sequencing of six different stages inflorescence samples. We emphasized on analyzing the modules highly associated with early and late reproductive stages to generate core coexpression networks underlying the inflorescence architecture using weighted gene co-expression network analysis (WGCNA). Based on the WGCNA results, we speculate that the *B3* (110723560) in the Green module and the *LFY* (110698679) in the Pink module are the most important regulators for the induction and maintenance of non-branching inflorescence, whereas the *MYB* (novel.11405) in the Blue module, the M-type *MADS* (novel.4708) in the Black module and the *TFL* (110697084) in the Turquoise module are essential for the inflorescence branching at late reproductive phase. Overall, the data obtained from this study will significantly contribute to our understanding of the networks regulating quinoa inflorescence architecture, as well will supply with valuable genetic resources for molecular breeding in the future.

## Methods

### Plant materials and growth conditions

Quinoa (*Chenopodium quinoa* Willd.) cultivator “LL-1” was used in this study. LL-1 was derived from a Bolivia quinoa cultivar “Puno” and was self-pollinated for at least six generations in the Experimental Station of Key Laboratory of Coarse Cereal Processing, Ministry of Agriculture in Jintang, Chengdu. The quinoa seeds were sown in a pot with 20 cm (cm) in diameter and 18 cm in depth. The sowing dates were July 19th, July 29th, August 24th, September 2nd, September 9th and September 16th in 2017. Seven days after sowing, the most robust five shoots in each pot were retained by removing the spared ones. All the quinoa plants were maintained in a greenhouse at Chengdu University N30^。^39′, E104^。^11′) with regular management, supplied with 200 mg/L water-soluble compound fertilizer (N:P_2_O_5_:K_2_O = 16:7:22) every 3 weeks and watered every 2 days. The quinoa plants were grown under natural photoperiod conditions which were approximately 13.8 h, 13.2 h, 12.3 h, 11.4 h, 10.6 h and 10.1 h in the middle of July, August, September, October, November and December in 2017, respectively. We manually removed the bracts using scalpel once a week to observe whether the plants had born floral buds. The young inflorescences (sown on August 24th, September 2nd, September 9th and September 16th in 2017, respectively referred to as YP4, YP3, YP2 and YP1) of five plants and the late-stage inflorescences (sowed on July 19th and July 29th, respectively referred to as P2 and P1) of three plants were collected on December 15th, three biological replicates for each occasion. The harvested samples were immediately stored in liquid nitrogen before total RNA extraction.

### RNA extraction and library preparation for high throughput sequencing

Total RNA of the samples collected above was extracted by using TRIzol reagent (Invitrogen) according to the manufacture’s instruction. The RNA concentration, purity and integrity were measured using Qubit® RNA Assay Kit in Qubit®2.0 Flurometer (Life Technologies, CA, USA), NanoPhotometer® spectrophotometer (IMPLEN, CA, USA) and the RNA Nano 6000 Assay Kit of the Bioanalyzer 2100 system (Agilent Technologies, CA, USA), respectively. The RNA samples whose RIN (RNA Integrity Number) values larger than 8.5 were used for following sequencing library construction. By applying poly-T oligo-attached magnetic beads, the mRNA from 3μg total RNA were isolated and purified. After fragmentation and double-strand cDNA synthesis, sequencing libraries were constructed using NEBNext® UltraTM RNA Library Prep Kit for Illumina® (NEB, USA) following the manufacturer’s protocols.

### RNA sequencing and data analysis

RNA sequencing was performed on an Illumina platform, and approximately 22 million to 31 million of paired-end 150 bp raw reads were generated. After removing the adaptor, low-quality and poly-N containing reads from raw reads, clean reads were obtained and used for downstream data analysis. Meanwhile, Q20, Q30 and GC content of the clean reads for each sample were calculated. Using HISAT (v2.0.5) [47] with default parameters, paired-end clean reads were mapped to the *Chenopodium quinoa* reference genome sequences [42] (http://ftp.ncbi.nlm.nih.gov/genomes/all/GCF/001/683/475/GCF_001683475.1_ASM168347v1). At the same time, StringTie (v1.3.3b) [48] was adopted to predict novel transcripts based on a novel network flow algorithm as well as an optional de novo assembly step. Subsequently, the novel genes were annotated through Pfam database [70]. For gene expression level analysis, Feature Counts (v1.5.0-p3) [71] was applied to count the reads numbers mapped to each gene, and FPKM (Fragments Per Kilobase of transcript sequence per Millions base pairs sequenced) value of each gene was calculated [49]. The Pearson correlations between biological replicates were calculated based on the FPKM values using the R function cor, and the result showed that all gene expression levels between replicates were highly correlated (R2 > 0.916). DESeq2 R package (1.16.1) [50] was used to normalize the read count value and dig out the differentially expressed genes (DEGs) between the groups (each with three biological replicates) at different developmental stages. The resulting *P* values were adjusted using the Benjamini and Hochberg’s approach for controlling the false discovery rate. An adjusted *P*-value less than 0.05 was applied to call DEGs between different groups.

### GO and KEGG enrichment analysis of DEGs

To understand the potential biological functions of DEGs, we used cluster Profiler R package to carry out Gene Ontology (GO) [72] and Kyoto Encyclopedia of Genes and Genomes (KEGG) [73] enrichment analysis. GO or KEGG terms with corrected *P*-value less than 0.05 were considered significantly enriched by DEGs.

### Transcription factor prediction of DEGs

To identify transcription factor encoding genes among DEGs, we adopted the Transcription Factor Prediction tool in Plant Transcription Factor Database v4.0 [51] (http://planttfdb.cbi.pku.edu.cn). ESTScan [74] was employed to identify and translate the Coding Sequence regions of the uploaded nucleic acid sequences. The defaulted parameters for TF family assignment and thresholds were adopted to identify transcrption factors from the DEG sequences.

### Construction of core regulatory network

All the DEGs were used to construct gene co-expression networks based on the expression levels (FPKM) by applying the Weighted Gene Co-Expression Network Analysis (WGCNA) [52] package in R. With default parameters (unsigned-type topological overlap matrix (TOM), power β was set to 6, minimal module size was set to 30, merge cut height was set to 0.25), the automatic network construction and module detection method were used for gene co-expression network construction. The edge weight (ranging from 0 to 1) which indicates the strength of the connectivity between two genes was calculated. Then, 21,568 out of 21,610 DEGs were clustered in 10 different modules. For core network construction, edge weight thresholds of 0.5, 0.3, 0.58, 0.55, and 0.58 were applied for refining the Green, Pink, Blue, Black and Turquoise module (Additional file 12: Dataset 1). Subsequently, TF encoding genes and key inflorescence architecture regulators in each module were used as seed nodes to generate core transcriptional regulatory network. Then, the core networks were visualized and analyzed using Cytoscape [53], and the genes with top connective degrees were defined as central hubs.

### Real-time PCR

The total RNA of each sample was extracted using TRIzol reagent. Two micrograms of total RNA for each sample were used for DNA digestion and reverse transcription to synthesize first-strand cDNA using EasyScript One-Step gDNA Removal and cDNA Synthesis SuperMix Kit (Transgen Biotech, Beijing, China). Subsequently, the properly diluted cDNA was used to perform real-time PCR by applying TransStart Green qPCR SuperMix (Transgen Biotech) on a real-time PCR system (LineGene 9600, BIOER, Hangzhou, China). The real-time PCR program was: 94 °C for 5 min, 39 cycles of denaturing at 95 °C for 5 s, annealing and extension at 59 °C for 20 s. A quinoa *MON* gene [75] (Monensin Sensitivity 1, Gene ID: 110720838) was used as the reference gene. The relative expression level of detected genes was quantified by the 2^−△△Ct^ algorithm from three replicates using Microsoft Office Excel 2016 (Washington, USA). The related primers used for real-time PCR were list in Additional file 11: Table S9. Then, the two-scale expression values (RNA-seq (FPKM) and real-time PCR) was normalized to be identical. For *CqUFO*/*APO1–1*, *APO1–2*, *CqTFL7* and *CqFTL9*, the gene expression values in YP3 obtained by real-time PCR and RNA-seq were both normalized to 1, and then the expression levels in other points (YP1, YP2, YP4, P1 and P2) were calculated according to the fold changes. For *CqFTL5*, the expression levels were calculated with the expression value in YP4 normalized to 1.

## Additional files

Additional file 1: **Figure S1.** Correlation coefficient between three biological replicates or between samples collected at different stages. The value was calculated based on the gene expression profiles using the R function cor. (DOCX 2439 kb)

Additional file 2: **Figure S2.** The TF percentages in DEGs and in quinoa genome. The TFs were identified using the Transcription Factor Prediction tool in Plant Transcription Factor Database v4.0, and then the proportion of TFs in DEGs (A) and the TF family percentages in quinoa genome (B) was calculated. (DOCX 1822 kb)

Additional file 3: **Table S1.** Statistic of the expressed genes at each stage. (DOCX 19 kb)

Additional file 4: **Table S2.** Statistic of the DEGs number between different samples. (DOCX 19 kb)

Additional file 5: **Table S3.** GO enrichment analysis of the DEGs at different intervals. (XLSX 50 kb)

Additional file 6: **Table S4.** Transcription factors identified in DEGs at different intervals. (XLSX 16 kb)

Additional file 7: **Table S5.** List of Transcription factors in quinoa genome. (XLSX 153 kb)

Additional file 8: **Table S6.** Genes distributed in different modules. (XLSX 5751 kb)

Additional file 9: **Table S7.** Transcription factors identified in different modules. (XLSX 33 kb)

Additional file 10: **Table S8.** The key genes used as seed nodes to generate core transcriptional regulatory network. (XLSX 16 kb)

Additional file 11: **Table S9.** The primer sequences used for real-time PCR in this study. (DOCX 17 kb)

Additional file 12: **Dataset 1.** The sequences of novel genes identified in this study. (TXT 41244 kb)

Additional file 13: **Dataset 2.** List of nodes included in different modules with stringent edge weight thresholds used for construction of gene regulatory networks. (XLSX 2433 kb)

## Data Availability

The RNA-seq data generated from *Chenododium quinoa* inflorescence samples in this study are deposited at NCBI SRA database (http://trace.ncbi.nlm.nih.gov/Traces/sra) under accessions SRR8361800, SRR8361801, SRR8361802, SRR8361803, SRR8361804 and SRR8361805.

## Abbreviations

AGL24: AGAMOUS-LIKE 24
ALOG: Arabidopsis LSH1 and Oryza G1
AMs: Axillary meristems
AP1: APETALA 1
APO1: ABERRANT PANICLE ORGANIZATION 1
BD1: BRANCHED SILKLESS1
BFL1: BRANCHED FLORETLESS 1
BMs: Branch meristems
BRC1: BRANCHED1
CLE: CLV3/ESR-related
CLV: CLAVATA
CO: CONSTANS
DAS: Days after sowing
DEGs: Differentially expressed genes
ERF: Ethylene-response factor
FEA2: FASCIATED EAR 2
FM: Floral meristems
FON1: FLORAL ORGAN NUMBER 1
FPKM: Fragments Per Kilobase of transcript sequence per Millions base pairs sequenced
FZP: FRIZZY PANICLE
GO: Gene ontology
IDS1: INDETERMINATE SPIKELET 1
IM: Inflorescence meristem
IPA1: Ideal Plant Architecture 1
KEGG: Kyoto Encyclopedia of Genes and Genomes
KN1: KNOTTED 1
KNOX: KNOTTED 1-like homeobox
LFY: LEAFY
MFS1: MULTI-FLORET SPIKELET 1
MOC1: MONOCULM 1
MOS1: MORE SPIKELETS 1
OSH1: *Oryza sativa* HOMEOBOX 1
PAP2: PANICLE PHYTOMER 2
PEBP: Phosphatidylethanolamine-binding protein
QTL: Quantitative trait loci
RA1: RAMOSA1
RCN1: REDUCED CULM NUMBER 1
RFL: RICE FLORICAULA
SAM: Shoot apical meristem
SBP: SQUAMOSA promoter binding protein-like
SEP: SEPALLATA
SNB: SUPERNUMERARY BRACT
SOC1: SUPPRESSOR OF OVEREXPRESSION OF CONSTANS 1
STM: SHOOT MERISTEMLESS
SVP: SHORT VEGETATIVE PHASE
TCP: TEOSINTE BRANCHED, CYCLOIDEA and PCF
TD1: THICK TASSEL DWARF 1
TF: Transcription factor
TFL1: TERMINAL FLOWER 1
TMF: TERMINATING FLOWER
TSH4: TASSELSHEATH4
TWA1: TAWAWA1
UFO: UNUSUAL FLORAL ORGAN
WFP: Wealthy Farmer’s Panicle
WGCNA: Weighted Gene Co-Expression Network Analysis
WOX: WUSCHEL-related homeobox
WUS: WUSCHEL
ZCN1: CENTRORADIALIS 1
