# Supplementary material for: Investigation into the underlying regulatory mechanisms shaping inflorescence architecture in Chenopodium quinoa
Source: BMC Genomics. 2019 Aug 17;20:658. doi: 10.1186/s12864-019-6027-0 (PMC6698048; doi:10.1186/s12864-019-6027-0)
Supplement: Supplementary file 1 — Figure S1. Correlation coefficient between three biological replicates or between samples collected at different stages. The value was calculated based on the gene expression profiles using the R function cor. (DOCX 2439 kb) [file 12864_2019_6027_MOESM1_ESM.docx]

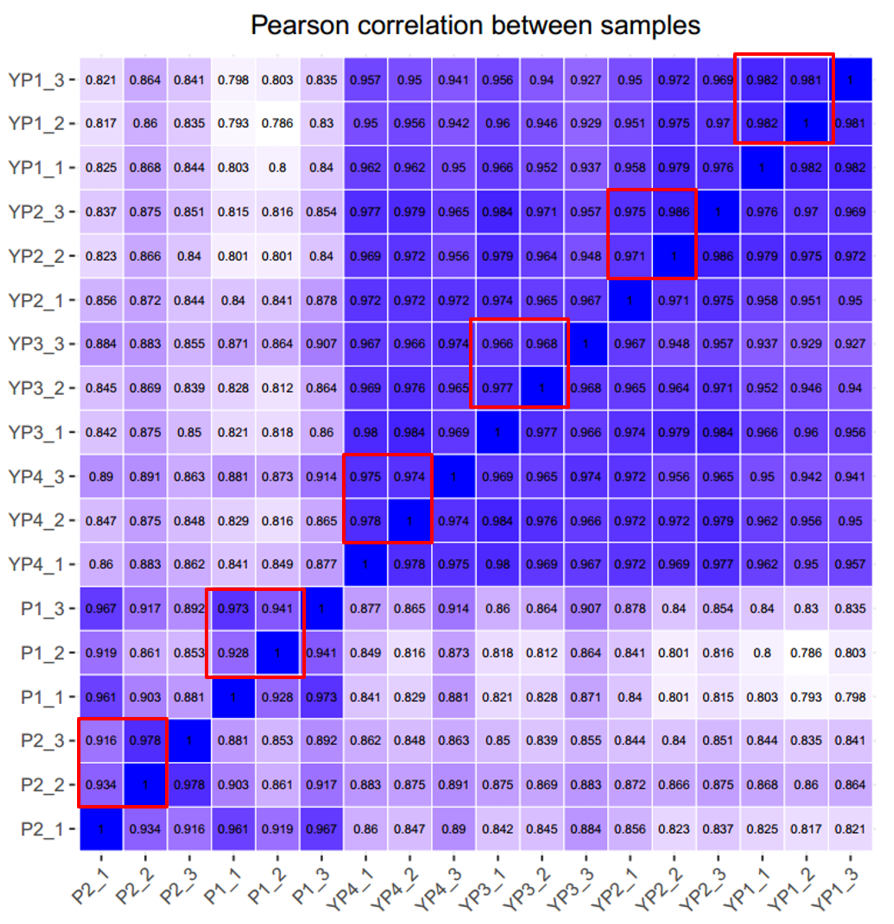


**Fig. S1** Correlation coefficient between three biological replicates or between samples collected at different stages. The value was calculated based on the gene expression profiles using the R function cor.
