# Supplementary material for: Investigation into the underlying regulatory mechanisms shaping inflorescence architecture in Chenopodium quinoa
Source: BMC Genomics. 2019 Aug 17;20:658. doi: 10.1186/s12864-019-6027-0 (PMC6698048; doi:10.1186/s12864-019-6027-0)
Supplement: Supplementary file 2 — Figure S2. The TF percentages in DEGs and in quinoa genome. The TFs were identified using the Transcription Factor Prediction tool in Plant Transcription Factor Database v4.0, and then the proportion of TFs in DEGs (A) and the TF family percentages in quinoa genome (B) was calculated. (DOCX 1822 kb) [file 12864_2019_6027_MOESM2_ESM.docx]

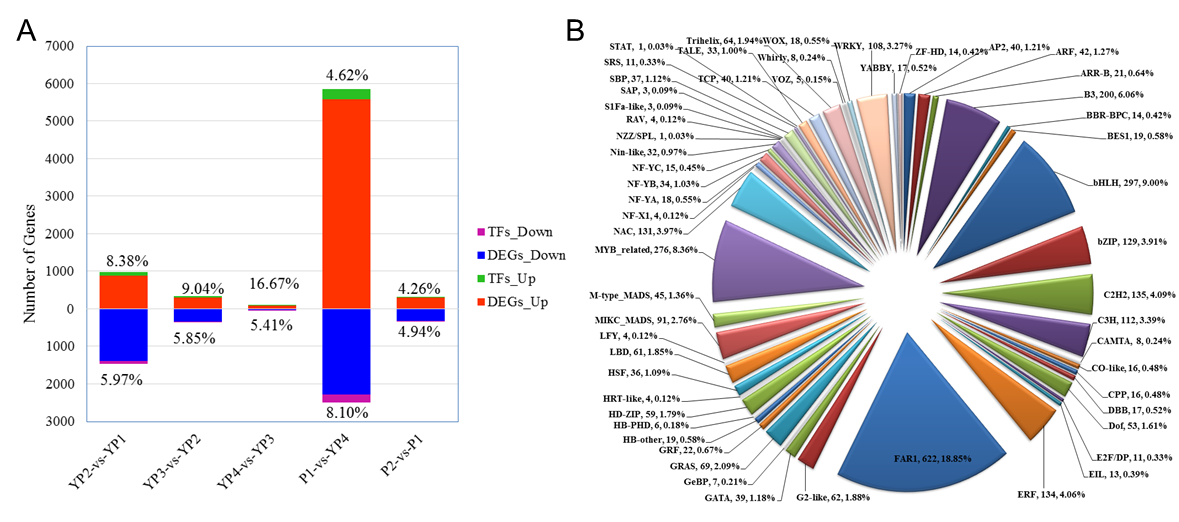


**Fig. S2** The TF percentages in DEGs and in quinoa genome. The TFs were identified using the Transcription Factor Prediction tool in Plant Transcription Factor Database v4.0, and then the proportion of TFs in DEGs (A) and the TF family percentages in quinoa genome (B) was calculated.
