# Supplementary material for: Investigation into the underlying regulatory mechanisms shaping inflorescence architecture in Chenopodium quinoa
Source: BMC Genomics. 2019 Aug 17;20:658. doi: 10.1186/s12864-019-6027-0 (PMC6698048; doi:10.1186/s12864-019-6027-0)
Supplement: Supplementary file 3 — Table S1. Statistic of the expressed genes at each stage. (DOCX 19 kb) [file 12864_2019_6027_MOESM3_ESM.docx]

| **Samples** | **YP1** | **YP2** | **YP3** | **YP4** | **P1** | **P2** |
| --- | --- | --- | --- | --- | --- | --- |
| **Number of Expressed genes(FPKM>1)** | 23951 | 24426 | 24794 | 24786 | 26770 | 25592 |

**Table S1 Statistic of the expressed genes at each stage.**
