# Supplementary material for: Investigation into the underlying regulatory mechanisms shaping inflorescence architecture in Chenopodium quinoa
Source: BMC Genomics. 2019 Aug 17;20:658. doi: 10.1186/s12864-019-6027-0 (PMC6698048; doi:10.1186/s12864-019-6027-0)
Supplement: Supplementary file 4 — Table S2. Statistic of the DEGs number between different samples. (DOCX 19 kb) [file 12864_2019_6027_MOESM4_ESM.docx]

**Table S2 Statistic of the DEGs number between different samples.**

| **Comparison** | **All DEGs** | **Up-regulated**  **DEGs** | **Down-regulated**  **DEGs** |
| --- | --- | --- | --- |
| YP2vsYP1 | 2441 | 967 | 1474 |
| YP3vsYP1 | 5242 | 2484 | 2758 |
| YP4vsYP1 | 4215 | 2145 | 2070 |
| P1vsYP1 | 11748 | 7792 | 3956 |
| P2vsYP1 | 13356 | 7593 | 5763 |
| YP3vsYP2 | 691 | 332 | 359 |
| YP4vsYP2 | 693 | 395 | 298 |
| P1vsYP2 | 10707 | 7265 | 3442 |
| P2vsYP2 | 12845 | 7358 | 5487 |
| YP4vsYP3 | 145 | 108 | 37 |
| P1vsYP3 | 8621 | 6019 | 2602 |
| P2vsYP3 | 11487 | 6871 | 4616 |
| P1vsYP4 | 8335 | 5841 | 2494 |
| P2vsYP4 | 10770 | 6307 | 4463 |
| P2vsP1 | 649 | 305 | 344 |
