# Supplementary material for: Investigation into the underlying regulatory mechanisms shaping inflorescence architecture in Chenopodium quinoa
Source: BMC Genomics. 2019 Aug 17;20:658. doi: 10.1186/s12864-019-6027-0 (PMC6698048; doi:10.1186/s12864-019-6027-0)
Supplement: Supplementary file 11 — Table S9. The primer sequences used for real-time PCR in this study. (DOCX 17 kb) [file 12864_2019_6027_MOESM11_ESM.docx]

**Table S9 The primers sequences used for real-time PCR in this study**

| **Gene ID** | **Forward (5’-3’)** | **Reverse (5’-3’)** |
| --- | --- | --- |
| CqMON1  (110720838) | GTATTGGTGGTCCTTGTG | CTGCTGTGGTGTATTGATT |
| CqUFO/APO1-1  (110686960) | GGTTGGAAGAGGACAAAACATG | TGGAGAACAAAGCGACTGAG |
| CqUFO/APO1-2  (110737497) | GTTGAGAGCAAGGACAGACTC | TGTGGCATCCTCTCGATTTC |
| CqFTL5  (110701053) | AAGAGTTGAGATTGGAGGTGATG | AGGTTTGGATTACTTGGGCTAG |
| CqFTL9  (110697999) | GAGCTTAGACCCTCTCAAACTG | CTCTTTCGTTTGGATTGCCAG |
| CqTFL7  (110697084) | GTAGACCCAGATGCTCCAAG | CCAGGAATATCCGTCACAATCC |
